# A fluorous-phase oxygen optical nanosensor for mitigating redox-active microbial metabolite interference

**DOI:** 10.1039/d6an00043f

**Published:** 2026-06-15

**Authors:** John M. Branning, Brianna M. Ruff, Samuel C. Saccomano, Avi I. Flamholz, Dianne K. Newman, Kevin J. Cash

**Affiliations:** a Quantitative Biosciences and Engineering Program, Colorado School of Mines Golden CO USA kcash@mines.edu; b The MITRE Corporation Bedford Massachusetts USA; c Chemical and Biological Engineering Department, Colorado School of Mines Golden CO USA; d Laboratory of Environmental Microbiology, The Rockefeller University New York NY 10065 USA; e Division of Biology and Biological Engineering, California Institute of Technology Pasadena California USA; f Resnick Sustainability Institute, California Institute of Technology Pasadena California USA; g Division of Geological and Planetary Sciences, California Institute of Technology Pasadena California USA

## Abstract

We developed a fluorous-phase oxygen-sensitive nanosensor that mitigates quenching effects caused by redox-active microbial metabolites whose effective lipophilicity depends on local chemical conditions, notably pyocyanin. The design encapsulates the fluorinated near-infrared (NIR) oxygen-sensitive luminophore platinum(ii) *meso*-tetra(pentafluorophenyl)porphine (PtTFPP) within a fluorous-phase matrix that restricts pyocyanin access to the dye relative to conventional non-fluorous polymer matrices, thereby reducing interference. Encapsulation within a fluorous polymer-based nanoparticle matrix maintains the dye at a constant loading in biological samples and avoids the need for complex synthetic approaches. The resulting fluorous-phase optical nanosensors exhibited consistent and reversible oxygen measurements across a wide concentration range, with pyocyanin-induced interference substantially attenuated relative to reference polymeric nanosensors. This design provides a framework for future research into fluorous nanosensing technologies and their application in diverse and complex environments.

## Abstract 2

Fluorous-phase oxygen nanosensors reduce pyocyanin-induced quenching by limiting metabolite partitioning while preserving O_2_ sensitivity, enabling more stable and reliable oxygen measurements in redox-active biological environments.

## Graphical abstract

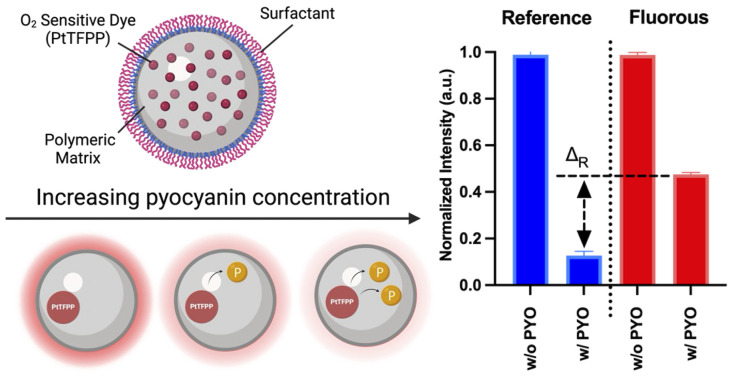

## Introduction

Oxygen serves as a crucial electron acceptor in cellular respiration across many biological systems. It is the preferred terminal electron acceptor for many organisms, but can also act as a potent reactive oxidant.^1–3^ Given its central role in physiological processes, numerous methods have been developed to measure oxygen concentrations in both natural environments and laboratory settings.^4–14^ A key application is the study of bacterial biofilms, which can form persistent infections in wounds and on medical implants and contribute to several chronic diseases, including cystic fibrosis.^15^ These biofilm-based infections are notoriously difficult to treat, as biofilms exhibit greater resistance to antibiotics compared to planktonic bacterial forms.^16^ Understanding the spatial and temporal metabolic variations in these biofilms is crucial for advancing treatment strategies. Recent enhancements in phosphorescent oxygen nanosensors now allow for the optical tracking of oxygen dynamics within bacterial biofilms.^17–20^

Nanoparticle-based sensors (nanosensors) are an emerging technology designed to address many challenges associated with biological measurements.^21–28^ One class of nanosensors consists of a highly plasticized polymer matrix that encapsulates one or more luminescent indicator dyes while allowing molecular oxygen to permeate the sensor.^26,29,30^ Many potential indicators exhibit poor solubility in water, which can pose difficulties in biological applications.^31^ Encapsulating these indicators within a polymer matrix improves control over the sensor's response and facilitates their use across a diverse range of samples. Moreover, these nanoparticles maintain stability over extended periods, ranging from hours to days, and can be uniformly distributed throughout the sample to measure three-dimensional gradients when used with appropriate optical techniques.^17^ Notable applications of oxygen-sensitive nanosensors include measurements of intracellular oxygen in cancer cells,^32–35^ monitoring oxygen dynamics in plants,^28,36^ and oxygen detection in microfluidic systems^37,38^ and *in vivo* measurements within bacterial biofilms.^18,39–42^

However, recent studies have demonstrated that redox-active molecules secreted by bacteria can interfere with these sensors,^43^ complicating accurate oxygen measurements in systems where these molecules are present. Experiments with purified substances revealed that this quenching arises from interactions with redox-active small molecules such as phenazines and flavins, which are produced by a wide range of microbial species in a density-dependent manner and can reach high concentrations.^44^ Pyocyanin, a defining phenazine produced by *Pseudomonas aeruginosa*, has been identified as a particularly strong quencher in these sensors.^43,45^ Importantly, the extent to which pyocyanin can access and accumulate within hydrophobic sensor matrices depends on local chemical conditions, including redox state and pH,^46^ which modulate its effective lipophilicity. As a result, phenazines such as pyocyanin may partition into the hydrophobic polymeric core of conventional oxygen sensors, enabling proximity-dependent quenching of embedded indicator dyes and complicating data interpretation in chemically complex environments.

**Fig. 1:**
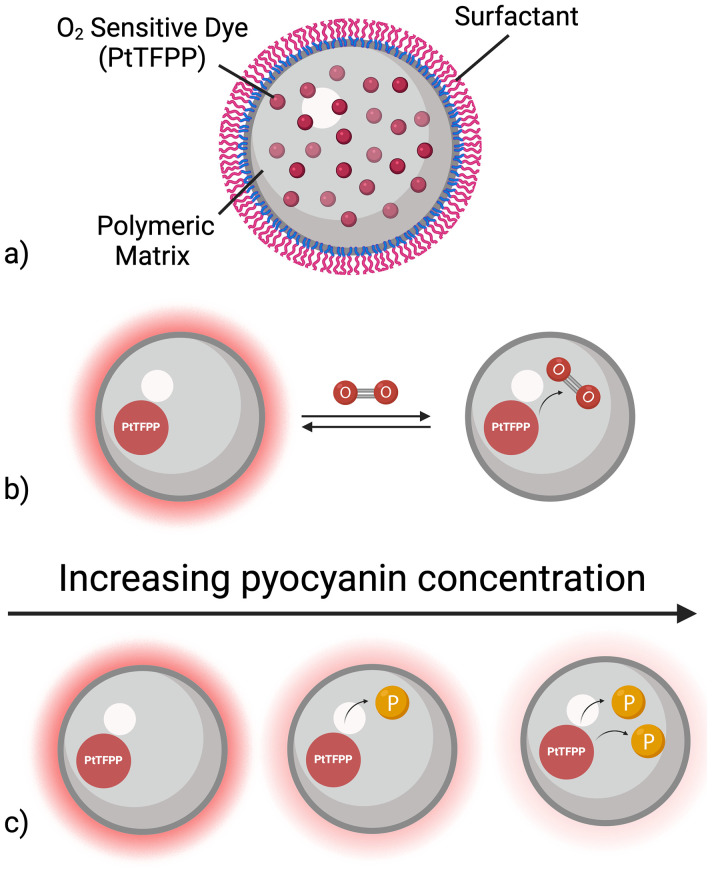
Conceptual design and sensing mechanism of fluorous oxygen nanosensors. (a) Schematic representation of nanosensors composed of the fluorinated oxygen-sensitive dye PtTFPP encapsulated within a fluorous polymer matrix. (b) Oxygen sensing mechanism. In the absence of O_2_, PtTFPP emits phosphorescence upon photoexcitation. In the presence of O_2_, collisional quenching of the excited triplet state occurs, dissipating energy through nonradiative pathways and reducing emission intensity as O_2_ concentration increases. (c) Pyocyanin-induced interference in conventional oxygen nanosensors, where increasing concentrations of pyocyanin quench nanosensor luminescence. Illustration created using BioRender (Branning, J., 2026). https://BioRender.com/gf4kwfw.

In optical nanosensors, the sensing phase typically consists of a polymer-plasticizer matrix that encapsulates the luminescent dyes, while a surfactant coating enables compatibility with aqueous environments (Fig. 1). The chemical composition of this matrix strongly influences sensor selectivity and dynamic range.^47–51^ Fluorinated compounds possess several distinctive physicochemical properties that are advantageous for nanosensor design.

Fluorous phases exhibit high oxygen solubility, enabling efficient oxygen transport to embedded dyes^48,52–55^ while their low affinity for polar and charged species limits the partitioning of many biological metabolites. This polarity mismatch reduces the accessibility of redox-active molecules, such as pyocyanin, to the sensing core, thereby mitigating metabolite-induced quenching. Fluorous polymers and surfactants also provide a favorable combination of permeability,^56–58^ selectivity,^59–61^ and chemical stability^62–64^ while remaining transparent through a wide ultraviolet, visible, and infrared spectral range, making them attractive candidates for nanosensor matrices.^65,66^

Fluorous materials have previously been used in ion-selective electrode sensors,^48,67–70^ fluorocarbon nanoemulsions for molecular imaging,^71^ and fluorous nanocomposite probes and sensors.^65,72,73–75^ Importantly, although elevated oxygen solubility supports efficient oxygen-dependent quenching, it does not inherently increase signal magnitude; rather, the primary advantage of fluorous phases lies in interference mitigation and environmental isolation.

Over the past decade, fluorinated variants of several dye and chromophore scaffolds, including coumarin, porphyrin, perylene, rhodamine, and BODIPY, have been developed.^54,76^ Fluorination of these structures often increases absorption coefficients and quantum yields, leading to advantageous properties such as red-shifted absorption maxima, enhanced brightness, and improved photostability.^54^ Platinum(ii) *meso*-tetra(pentafluorophenyl)porphine (PtTFPP), a fluorinated variant of platinum(ii) *meso*-tetraphenylporphine (PtTPP), is a luminescent dye that emits at *λ*_em_ = 650 nm when excited at *λ*_ex_ = 405 nm and is reversibly quenched by molecular oxygen, thus making it well suited for optical measurements of oxygen in biological samples.^77–79^

We therefore hypothesized that incorporating fluorous polymers together with fluorinated surfactants would reduce the phase transfer of secreted bacterial metabolites into the nanosensor core, thereby minimizing their confounding effects on oxygen-sensitive optical nanosensors. In this design, the surfactant layer stabilizes the nanoparticle in aqueous environments, while the fluorous polymer matrix selectively promotes the diffusion of O_2_ into the sensing region, where it interacts with the fluorinated dye.

## Experimental

### Materials

High-molecular-weight poly(vinyl chloride) (PVC), poly(vinylidene fluoride) (PVDF), bis(2-ethylhexyl) sebacate (BEHS), tetrahydrofuran (THF), dichloromethane (DCM), and phosphate-buffered saline (PBS), poly[4,5-difluoro-2,2-bis(trifluoromethyl)-1,3-dioxole-*co*-tetrafluoroethylene] (PFPE, Teflon AF 2400), hexafluorobenzene (HFB), perfluoro(methylcyclohexane), α,α,α-trifluorotoluene (TFT), pyocyanin, α-d-glucose and glucose oxidase from *Aspergillus niger*, 0.22 µm sterile vacuum filter, 15- and 18-gauge needles were purchased from Sigma-Aldrich (St Louis, MI, USA). Platinum(ii) *meso*-tetra(pentafluorophenyl)porphine (PtTFPP) and palladium(ii) *meso*-tetra(pentafluorophenyl)porphine (PdTFPP) were purchased from Frontier Scientific (Logan, UT, USA). 1,2-Dipalmitoyl-*sn-glycero*-3-phosphoethanolamine-*N*-[methoxy(polyethylene glycol)-750] ammonium salt in chloroform (PEG-lipid) was purchased from Avanti Polar Lipids (Alabaster, AL, USA). 4-Di-16-ASP (4-[4-(dihexadecylamino)styryl]-*N*-methylpyridinium iodide) (DiA), and 96-well black-walled optical bottom plates were purchased from Thermo Fisher Scientific (Waltham, MA, USA). Ultrahigh purity nitrogen gas and compressed air were purchased from Matheson (Denver, CO, USA). 10 mm pathlength quartz cuvette with rubber septa seal cap was purchased from Starna Cells (Atascadero, CA, USA). Perfluoropolyether polyethylene glycol (PFPE-PEG, PFPE 5k, PEG 5k) was purchased from Creative PEGWorks (Chapel Hill, NC, USA). *Pseudomonas aeruginosa*, strain PA14, was purchased from BEI Resources (Manassas, VA, USA).

### Methods

#### Fabrication of nanosensors

To fabricate the non-fluorous nanosensor we first fabricated the non-fluorous optode formulation with the following sensor components. 15 milligrams of PVC was weighed out into a 2 mL glass vial and combined with 33 µL of BEHS, followed by vortex mixing. 2.5 mg of PtTFPP and 0.2 mg of DiA were dissolved in 250 µL of THF and transferred to the PVC slurry. The mixture was vortexed until the PVC was completely dissolved. Subsequently, 375 µL of DCM was added to the vial containing the PVC–dye mixture. The optode solution was then capped and stored at 4 °C until further use.

To prepare the nanosensors, 80 µL of PEG-750 (25 mg mL^−1^ in chloroform) was added to a 4-dram scintillation vial. The chloroform was evaporated under a gentle air stream, after which the PEG-750 residue was redissolved in 5 mL of PBS and sonicated with a probe-tip sonicator (Branson Ultrasonics, Brookfield, CT, USA) for 30 s at 20% amplitude. Subsequently, 125 µL of the optode solution was added, and the mixture was sonicated for another 3 minutes at 20% amplitude to form the nanosensors. The resulting nanosensor suspension was filtered through a 0.8 µm polyethersulfone membrane into a 5 mL glass vial and stored at room temperature protected from light.

To fabricate the fluorous nanosensor we first fabricated the fluorous optode formulation with the following sensor components. 15 milligrams of PVDF was weighed out into a 2 mL glass vial and combined with 33 µL of BEHS, followed by vortex mixing. 2.5 mg of PtTFPP was dissolved in 250 µL of THF and transferred to the PVDF slurry. The mixture was vortexed until the PVDF was completely dissolved. Subsequently, 375 µL of DCM was added to the vial containing the PVDF–dye mixture. The optode solution was then capped and stored at 4 °C until further use.

To prepare the nanosensors, 80 µL of PFPE-PEG (25 mg mL^−1^ in THF) was added to a 4-dram scintillation vial. The THF was evaporated under a gentle air stream, after which the PFPE-PEG residue was redissolved in 5 mL of PBS and sonicated with a probe-tip sonicator (Branson Ultrasonics, Brookfield, CT, USA) for 30 s at 20% amplitude. Subsequently, 125 µL of the optode solution was added, and the mixture was sonicated for another 3 minutes at 20% amplitude to form the nanosensors. The resulting nanosensor suspension was filtered through a 0.8 µm polyethersulfone membrane into a 5 mL glass vial and stored at room temperature protected from light.

#### Gas flow and oxygen sensor characterization

Calibration of the oxygen sensors was done using a gas flow system of mixed ultrapure nitrogen and air streams.^79^ Three milliliters of nanosensor suspension was transferred to a quartz cuvette (1 cm pathlength) sealed with a rubber septum cap. Luminescence measurements were acquired with an Avantes Avaspec-2408L spectrometer with excitation from fiber-coupled LED (*λ*_ex_ = 405 nm; ThorLabs, Newton, NJ, USA).

Nitrogen and air flow rates were regulated using two mass flow controllers (Alicat Scientific, Tucson, AZ, USA). The gas streams were then mixed in a 50 mL stainless-steel gas mixing chamber before entering the cuvette.^79^ The gas mixture was bubbled directly into the cuvette through the septum seal using a 15-gauge needle attached to the gas line. A second needle was inserted through the septum to allow excess gas to vent from the cuvette during bubbling.

The total gas flow rate was maintained at 5 mL min^−1^, while the relative flow rates of nitrogen and air were adjusted to achieve oxygen concentration between 0% and 21% in the gas phase. Measurements were obtained at 0%, 5%, 10%, 15% and 21% O_2_ in the gas phase, which was equivalent to 0, 1.58, 3.17, 4.75 and 6.65 mg L^−1^, respectively, confirmed with microelectrode measurements (UniSense, Aarhus, DNK). The initial equilibration period at 0% O_2_ was 45 min, while subsequent equilibration steps were 45 min.

A calibration curve was generated by measuring the intensity at *λ*_em_ = 650 nm (PtTFPP emission) at each oxygen concentration. The data were analyzed using the Stern–Volmer relationship, expressed as a ratio of the intensity in the absence quencher (*I*_0_, 0 mg L^−1^ O_2_) to the intensity at a given concentration (*I*). The Stern–Volmer constant (*K*_SV_) was determined by linear regression of the Stern–Volmer plot, as described by eqn (1) in the Results section.

Nanosensor reversibility was evaluated by alternating between pure nitrogen (deoxygenation) and air (21% O_2_) to determine whether the luminescence response changed as a function cycling. Each bubbling step was maintained for 45 min, between measurements, and the experiment was repeated for 5 cycles.

The pH response was assessed by diluting the sensor suspension 1 : 1 with potassium buffer solutions prepared from phosphate dibasic and potassium phosphate monobasic adjusted to different pH values.

#### Pyocyanin response characterization

The effect of the pyocyanin on the response of the oxygen nanosensors was evaluated using a glucose/glucose oxidase reaction to scavenge dissolved oxygen and generate an anoxic environment in the solution. Deoxygenated samples were prepared using 100 μM glucose and 20 IU mL^−1^ glucose oxidase stocks solutions in PBS. Each well contained 100 μL of nanosensors. For deoxygenated conditions, 50 μL each of the glucose and glucose oxidase stocks were added. In samples containing pyocyanin, 50 μL of 100 μM pyocyanin dissolved in PBS were added, where as control samples without pyocyanin received 50 μL of PBS. Oxygenated controls contained PBS only, glucose with PBS, or glucose oxidase with PBS, with the total well volume maintained at 250 μL. Luminescence measurements were performed on a Synergy H1 microplate reader (Biotek, Winooski, VT, USA). Signals were collected from the top of the plate with a gain of 100 using excitation at *λ*_ex_ = 405 nm and emission detection at *λ*_em_ = 650 nm.

## Results

**Fig. 2:**
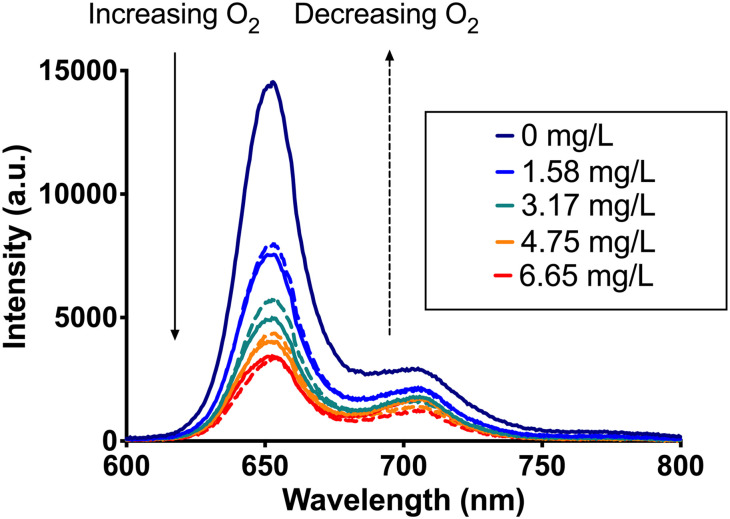
Luminescence spectra of fluorous nanosensors containing PtTFPP. Emission spectra were recorded with excitation at *λ*_ex_ = 405 nm using an LED light source. The oxygen-sensitive emission peak occurs at *λ*_em_ ≈ 650 nm. Dissolved oxygen concentration was controlled by bubbling mixtures of air and nitrogen. Luminescence spectra (solid lines) were recorded from anoxic conditions (0 mg L^−1^ O_2_) to near-atmospheric oxygen levels (6.65 mg L^−1^ O_2_, corresponding to atmospheric equilibrium at 5675 ft elevation in Golden, CO). Dashed lines represent reversibility measurements collected during sequential oxygenation and deoxygenation cycles.

We fabricated fluorous oxygen-sensitive nanosensors by encapsulating a dye (PtTFPP) in a fluorous nanoparticle matrix. The dye was chosen because it is a fluorinated oxygen sensitive dye which was previously used in nanosensors.^39,80,81^ The PtTFPP luminescence will change based on the quenching of oxygen, meaning that higher oxygen concentrations correlate to a lower observed emission intensity when the dye is excited. Fig. 2 shows the luminescence spectra of the fluorous nanosensor. In these spectra, we can see a clear response of the oxygen dye as the concentration of dissolved oxygen in the sample is changed.

The quantum yield^82^ of PtTFPP within the fluorous nanosensor (*Φ* = 0.070 deoxygenated, Fig. S1) is within the range of values reported for PtTFPP-based nanosensors^77,83–88^ (Table S1). The lifetime of the fluorous nanosensors measured using the Rapid Lifetime Determination (RLD) method,^89,90^*τ*_0_ = 63–69 μs, is approximately 1.8× longer than the reference nanosensors^31,48,54,91–95^ (Table S2). The radiative rate constant *k*_R_ = *Φ*/*τ*_0_ = 0.0011 μs^−1^ and total non-radiative rate constant *k*_NR_ = (1 − *Φ*)/*τ*_0_ = 0.0135 μs^−1^ were estimated using the midpoint *τ*_0_ value.^96,97^

In luminescence quenching-based methods, the Stern–Volmer equation (eqn (1)) describes the relationship between the quencher concentration (here [O_2_]) and signal intensity.1
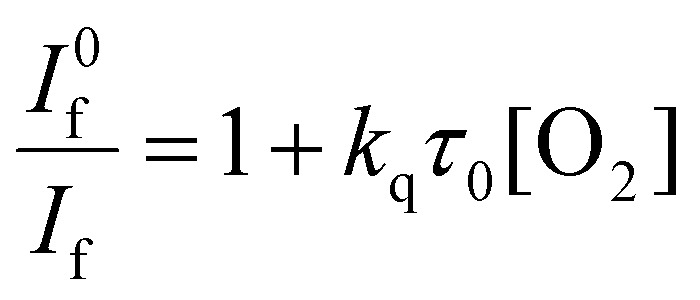
*I*^0^_f_ and *I*_f_ represent the luminescence signal intensity in the absence and presence of oxygen at a given concentration, respectively. The quenching constant (*k*_q_) and the unquenched decay lifetime (*τ*_0_) combined to form the Stern–Volmer constant, *K*_SV_.

**Fig. 3:**
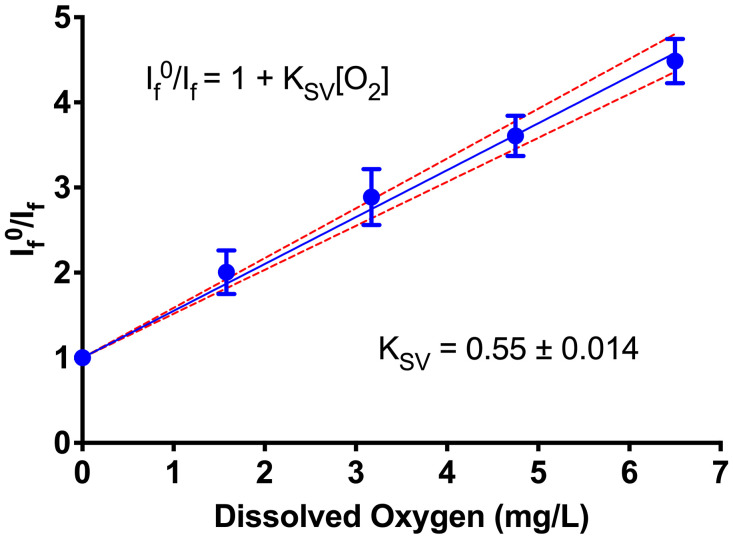
Stern–Volmer calibration of fluorous oxygen nanosensors. The normalized luminescence response is plotted as a function of dissolved oxygen concentration. The Stern–Volmer relationship remained linear over the tested range of 0–6.35 mg L^−1^ O_2_. The Stern–Volmer constant (*K*_SV_) corresponds to the slope of the linear regression with *R*^2^ = 0.98. Error bars represent the standard deviation of replicate measurements (*n* = 3), and the dotted lines indicate the 95% confidence interval of the linear regression.

We created Stern–Volmer plots (Fig. 3) by measuring the luminescence intensity of PtTFPP as a function of [O_2_]. The Stern–Volmer constant (*K*_SV_ = 0.55 ± 0.014 L mg^−1^) was determined by linear regression of the Stern–Volmer plot, as described by eqn (1). This value quantifies the degree to which the nanosensor-embedded PtTFPP is quenched by O_2_, with larger values indicating stronger quenching. Given this *K*_SV_, luminescence intensity is roughly fourfold higher in anoxic conditions (0%) than air-levels of O_2_ (21%).

To monitor oxygen concentrations in biological systems, sensors must be reversible, *i.e.* able to recover luminescence after transient O_2_ exposure. To test reversibility of the nanosensors, we measured the linearity of the Stern–Volmer response during deoxygenation and reoxygenation by alternately bubbling the same sample with nitrogen and air mixtures. The oxygen concentration was varied in 5% increments from 0% to 21%, and then back from 21% to 0%, to assess consistency in both directions. The signal was measured over two complete cycles between oxygenated and deoxygenated conditions. No statistical difference in *K*_SV_ values was observed between the forward and reverse cycles (Fig. S2A), demonstrating that the nanosensor response is both reproducible and reversible. This behavior suggests that the curvature observed in (Fig. 3 and Fig. S2) does not arise from irreversible or static quenching effects. Instead, residual nonlinear response (Fig. S3) may reflect measurements acquired under non-equilibrium conditions during oxygen transitions.

The Stern–Volmer calibration was also performed for the reference polymeric nanosensors (Fig. S2B), revealing that the nanosensor systems do not exhibit equivalent intrinsic oxygen sensitivity. The observed difference in *K*_SV_ does not confound interpretation of the pyocyanin interference experiments because all measurements were performed under fully deoxygenated conditions generated using excess glucose and glucose oxidase. Under these conditions, oxygen-dependent quenching is absent and both nanosensor systems operate at their respective *I*_0_ baseline, such that subsequent signal attenuation reflects exclusively pyocyanin-induced quenching.

Beyond reversibility, practical deployment in biological environments requires robustness to physiochemical fluctuations such as pH variation. The fluorous nanosensors exhibited stable emission across the investigated pH range (3–9.5) under atmospheric oxygen conditions (Fig. S4). Although one-way ANOVA detected statistically significant differences in luminescence intensity across pH 3–9.5 (*F* = 10.28, *p* = 0.00019), the overall variation was small relative to the mean signal (∼4% coefficient of variation) and showed no monotonic pH-dependent trend, indicating that the nanosensors maintain functional emission stability across a physiologically relevant pH range.

In addition to environmental stability, resistance to redox-active metabolite interference represents a central challenge for oxygen sensing in microbial systems. We therefore evaluated the ability of fluorous nanosensors to resist pyocyanin quenching by measuring the luminescence response of the sensor to pyocyanin in a deoxygenated buffer generated by the addition of excess glucose and glucose oxidase (see Methods).

**Fig. 4:**
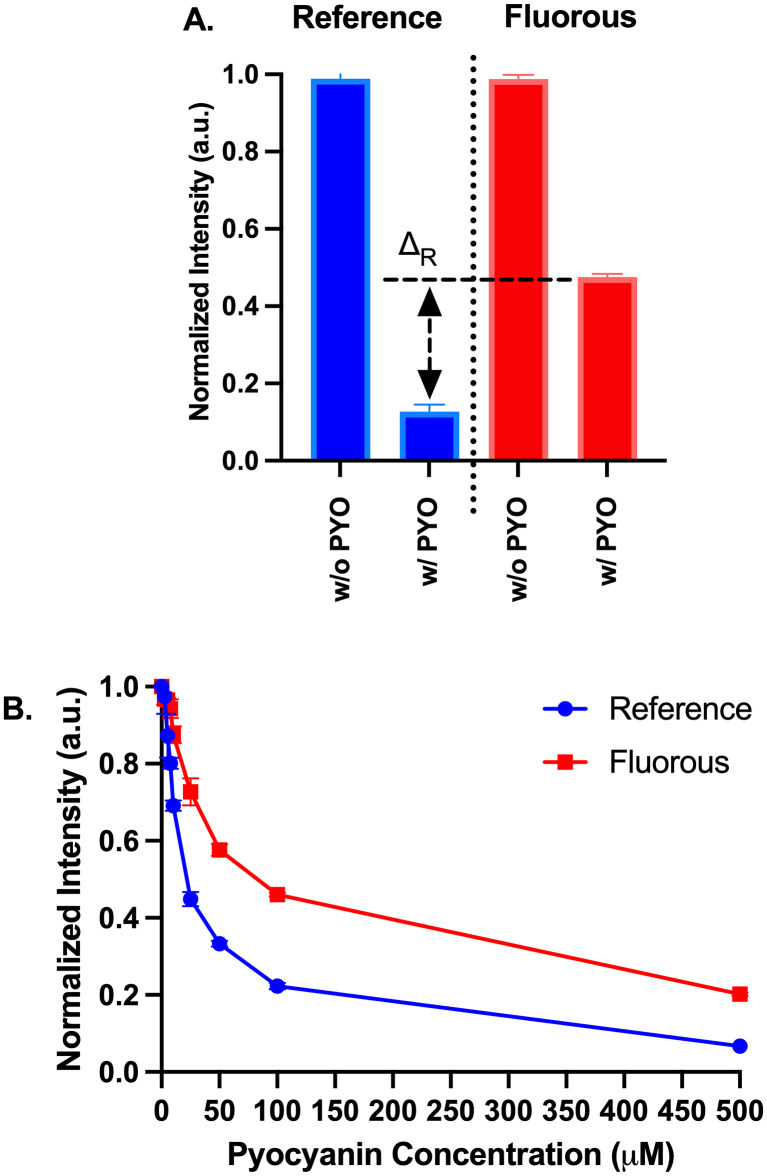
Pyocyanin-induced quenching of oxygen nanosensors. (A) Deoxygenated luminescence response of reference polymeric nanosensors and fluorous nanosensors following addition of 100 μM pyocyanin. Reference polymeric nanosensors exhibited an 80% reduction in deoxygenated luminescence, whereas fluorous nanosensors exhibited a 46% reduction. Error bars represent the standard deviation (*n* = 3). (B) Concentration-dependent pyocyanin quenching measured at 0, 2.5, 5.0, 7.5, 10, 25, 50, 100, and 500 μM pyocyanin. Fluorous nanosensors maintained higher normalized deoxygenated luminescence across all pyocyanin concentrations.

As shown in Fig. 4 fluorous nanosensors were less affected by pyocyanin quenching than earlier nanosensor designs lacking fluorous phases. The deoxygenated response of oxygen-sensitive nanosensors in the presence of 100 μM pyocyanin reveals significant differences: reference polymeric nanosensors showed an 80% reduction in deoxygenated luminescence, whereas fluorous nanosensors exhibited only a 46% reduction (Fig. 4A). The fluorous nanosensors outperformed the traditional nanosensors across all measured pyocyanin concentrations (Fig. 4B).

**Fig. 5:**
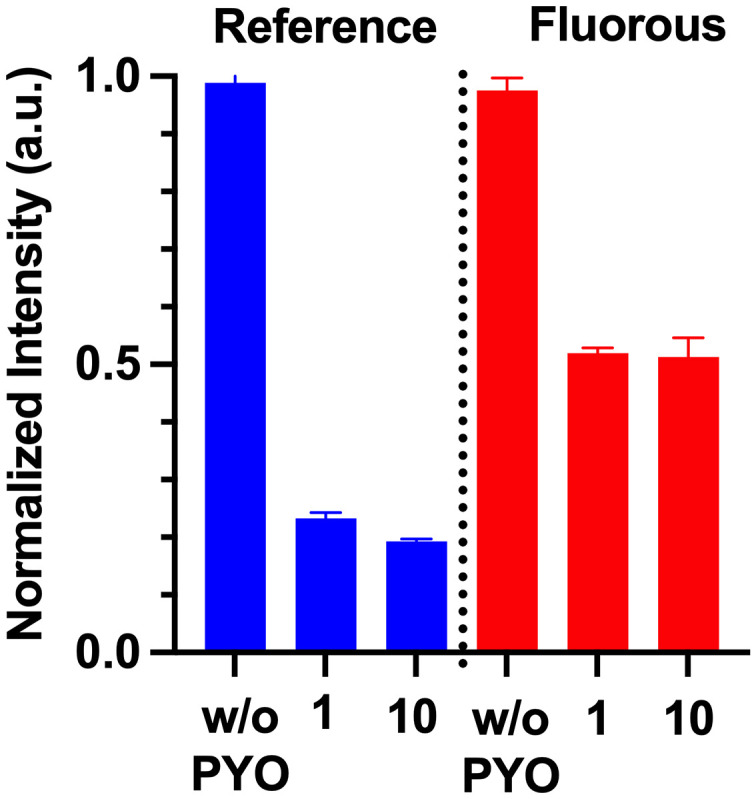
Long-term luminescence stability of nanosensors in the presence of pyocyanin. Luminescence responses of reference polymeric nanosensors and fluorous nanosensors were monitored over a 10 day period. 100 μM pyocyanin was added to one sample of each nanosensor type on day 1. The experiment was repeated on day 10 using freshly prepared samples drawn from the same nanosensor batches. Fluorous nanosensors maintained higher residual luminescence compared to reference nanosensors throughout the exposure period. Error bars represent the standard deviation (*n* = 3).

Lifetime performance of the nanosensors over a 10 day period shows that the reference nanosensors’ luminescence remains significantly quenched, whereas the fluorous nanosensors continue to exhibit relatively higher residual luminescence levels. Functional lifetime measurements (Fig. 5) indicate that fluorous-phase nanosensors confer durable protection against pyocyanin over 10 days, with only a 1.3% decrease in luminescence intensity compared to a 16.8% decrease observed for reference polymeric nanosensors (Fig. S5). This resilience results in more reliable oxygen measurements in environments where organic quenchers like pyocyanin are present, supporting the use of fluorous nanosensors for applications requiring long-term and stable oxygen sensing in complex biological systems such as bacterial biofilms.

## Discussion

In this study, we evaluated nanosensor performance using controlled abiotic and enzymatic model systems to isolate the influence of pyocyanin-mediated quenching. We demonstrate that specific fluorous nanosensor formulations can significantly reduce, though not eliminate, pyocyanin-induced quenching while preserving sensitivity to molecular oxygen. Across the formulations examined here, resistance to pyocyanin interference depended not only on polymer fluorination, but also on luminophore chemistry and fabrication conditions, indicating that interference mitigation is a formulation-dependent phenomenon rather than an intrinsic property of all fluorous matrices. Across all tested pyocyanin concentrations, fluorous nanosensors exhibited diminished pyocyanin-mediated quenching relative to traditional polymer-based designs, supporting the hypothesis that fluorous phases restrict access of weakly polar redox-active metabolites to the embedded dye.

Pyocyanin is a redox-active phenazine that participates in coupled electron/proton transfer reactions under physiological conditions and can undergo oxygen-coupled redox cycling in enzymatic and biological environments.^44,98–100^ In glucose and glucose oxidase enzymatic reactions, pyocyanin acts as an artificial electron acceptor,^101,102^ where oxidized pyocyanin is reduced enzymatically and subsequently re-oxidized by transferring electrons to oxygen. This cycling can sustain a population of reduced pyocyanin under conditions of active enzymatic turnover, particularly in low-oxygen or metabolically active environments.

The phosphorescent dye PtTFPP emits from a long-lived triplet excited state that is efficiently quenched by molecular oxygen through collisional interactions, but can also undergo additional nonradiative decay pathways in the presence of redox-active species.^97^ In conventional polymeric nanosensors, weakly polar molecules such as pyocyanin can partition into the hydrophobic polymer matrix, enabling proximity-dependent quenching of the embedded dye.^43^ Fluorous polymers, in contrast, are characterized by low polarizability and reduced solubility for many organic solutes. The dominant non-radiative decay in the fluorous formulation (*Φ*, *k*_NR_ ≫ *k*_R_) is consistent with published values for PtTFPP embedded in nanoparticle matrices, confirming that the fluorous matrix does not enhance dye brightness relative to the reference formulation.

Consistent with these properties, our data show that pyocyanin-induced quenching is substantially attenuated in fluorous nanosensors, indicating reduced molecular partitioning into the sensing phase and confirming that mitigation of oxygen-independent interference is the principal functional advantage of the fluorous matrix in biologically complex environments. However, the fluorous matrix does not provide complete exclusion, and measurable quenching persists under conditions that sustain redox cycling.

Importantly, expanded formulation screening revealed that fluorination of the polymer matrix alone is insufficient to guarantee improved resistance to pyocyanin interference. Several fluorinated formulations (Fig. S12–S15 and Table S6) exhibited pyocyanin-induced quenching comparable to or greater than conventional PVC-based reference systems. Likewise, incorporation of a non-fluorinated luminophore into a conventional PVC matrix did not improve resistance to pyocyanin quenching (Table S6). Collectively, these observations indicate that nanosensor performance depends on coupled interactions among polymer composition, luminophore physicochemical properties, nanosensor internal organization, and fabrication methodology.

Taken together, our results support a mechanistic framework in which pyocyanin-mediated quenching is governed by coupled redox-state and partitioning effects influenced by nanosensor microstructure and dye localization within the particle interior. The redox state of pyocyanin modulates its effective lipophilicity, with the reduced form exhibiting greater affinity for hydrophobic environments.^46^ This increased lipophilicity enhances partitioning into or near the nanosensor matrix, enabling proximity-dependent interactions with the luminophore. Under conditions that support sustained redox cycling, such as enzymatic reactions or biological systems, a persistent population of reduced pyocyanin can be maintained, increasing its effective local concentration and strengthening quenching. In contrast, under purely abiotic, oxygenated conditions, pyocyanin remains predominantly oxidized and exhibits reduced partitioning into the sensor matrix, resulting in weaker and less reproducible quenching.

Consistent with this framework, oxidized pyocyanin under abiotic, oxygenated conditions produced minimal and irreproducible quenching (see Fig. S6–S9), whereas stronger and more reproducible quenching was observed in glucose–glucose oxidase systems (Fig. 4 and 5). Although pH can influence pyocyanin protonation^45^ and, together with redox state, alter its effective lipophilicity,^46^ the pH range examined here did not measurably suppress quenching in conventional polymeric nanosensors (see Fig. S8). Instead, the presence of sustained redox cycling appears to be the dominant factor governing interference under the conditions tested. These observations indicate that pyocyanin interference is highly context dependent and arises from the interplay between solution-phase redox processes and matrix-level partitioning behavior.

The discrepancy between the weak abiotic quenching observed in this study and stronger abiotic effects reported previously suggests that pyocyanin-mediated interference is highly sensitive to local chemical conditions that control its redox state and effective partitioning. Even nominally abiotic systems may differ in oxygen availability, reducing capacity, and protonation conditions, leading to variations in the fraction of reduced pyocyanin and, consequently, its accessibility to the nanosensor matrix. These differences likely account for the variability in reported quenching behavior across studies.

While the present results are consistent with redox-state–dependent partitioning, this study does not directly quantify pyocyanin uptake into the fluorous phase nor independently measure the redox-state distribution within the nanosensor microenvironment. Consequently, we cannot definitively distinguish whether residual quenching arises primarily from limited metabolite penetration into the fluorous matrix, interfacial interactions near the nanosensor surface, or redox-mediated processes in the surrounding solution. Resolving these contributions will require future studies incorporating direct permeability measurements and controlled modulation of pyocyanin redox state.

The immiscibility between fluorous and conventional organic phases presents practical challenges for nanosensor fabrication,^103^ as many optode components are traditionally processed in organic solvents. Consistent with this limitation, fabrication of fluorous-phase nanosensors using conventional organic solvents resulted in pronounced batch-to-batch variability in sensor response (Fig. S10A–C). Hierarchical analysis of the coefficient of variation (Tables S4, S5 and Fig. S10A–D) indicates that variability between independently prepared batches exceeds the variability observed within individual nanosensor solutions. This pattern suggests that differences arising during nanosensor preparation contribute more strongly to signal dispersion than measurement-level noise. Formulation-dependent differences in pyocyanin resistance further indicate that fabrication conditions influence not only reproducibility, but also nanosensor transport properties and accessibility of the embedded luminophore to redox-active interferents. Dynamic light scattering (DLS) measurements further revealed moderate polydispersity, with average hydrodynamic diameter of 212 nm with a relatively broad size distribution (Fig. S11), consistent with non-uniform nanosensor formation. Although intra-batch precision remains high, these findings highlight batch reproducibility as a key practical consideration for translational applications and emphasize the importance of batch-specific calibration in biological measurements.

Systematic variation of fluorous polymers, solvents, and oxygen-sensitive dyes revealed substantial formulation-dependent differences in pyocyanin interference behavior (see Fig. S12–S15), suggesting that the current system may be limited by fundamental transport or interfacial constraints rather than specific material combinations. Nevertheless, fluorous nanosensors demonstrated reduced susceptibility to metabolite interference while maintaining robust oxygen sensitivity, indicating their potential utility in biofilm-relevant chemical conditions.

Although fluorous materials do not fully eliminate pyocyanin-induced quenching, they provide a meaningful reduction in interference and are expected to improve measurement robustness in biofilm-relevant conditions. Future work should focus on decoupling redox-state–dependent partitioning from solution-phase quenching mechanisms, for example through controlled electrochemical modulation of pyocyanin redox state. From an engineering perspective, the development of fully fluorous-processable nanosensor components and surfactants may enable fabrication in fluorous solvents, improving structural uniformity and reducing batch variability. Additionally, distinguishing whether residual signal loss arises from matrix partitioning, interfacial accumulation, or direct redox quenching will guide the rational design of nanosensor systems with enhanced selectivity in metabolically active environments.

## Conclusions

Although further refinement is needed, the development of fluorous-phase optical nanosensors for oxygen concentration sensing represents a meaningful step toward improving nanosensor stability and reliability in biological environments. In this study, we intentionally employed well-controlled abiotic and enzymatic model systems to isolate and quantify metabolite-induced quenching and to evaluate the extent to which the fluorous design mitigates this effect. Within these defined systems, the nanosensors demonstrated reduced quenching by pyocyanin. In the optimized fluorous formulation examined here, encapsulation of the oxygen-responsive dye within a fluorous matrix appears to limit partitioning of pyocyanin while preserving oxygen permeability, resulting in attenuated, but not fully eliminated, interference.

Our results further indicate that pyocyanin-mediated quenching is strongly dependent on chemical context, particularly conditions that sustain reduced, more lipophilic pyocyanin. As such, interference is expected to be amplified in metabolically active or redox-cycling environments, underscoring the importance of considering redox state when evaluating nanosensor performance. Accordingly, this work should be interpreted as demonstrating that fluorous-phase encapsulation represents a mechanistically grounded strategy for improving the resistance to metabolite interference, rather than a complete solution to this challenge in complex microbial systems.

Importantly, pyocyanin resistance emerged as a formulation-specific property governed by coupled effects of polymer chemistry, luminophore identity, nanosensor microstructure, and fabrication conditions. These findings define the current scope of applicability for fluorous encapsulation strategies and highlight the need for formulation-specific validation when extending this approach to other sensing systems.

This work contributes to the broader understanding of fluorous materials in optical nanosensing and establishes a framework for rational sensor design based on controlling molecular partitioning and access to the sensing domain. While further validation in fully biological and *in situ* systems is necessary, these results highlight the potential of fluorous encapsulation to improve robustness in biofilm-relevant environments. Future studies should quantify performance limits under dynamic biological conditions, evaluate long-term biofouling behavior, and determine the physicochemical criteria governing successful implementation of fluorous encapsulation across different fluorophores, matrices, and analyte systems.

## Author contributions

JMB: conceptualization, investigation, methodology, validation, visualization, writing – original draft, writing – review & editing. BMR: investigation, validation. SCS: investigation, methodology, validation. AIF: conceptualization, writing – review & editing. DKN: conceptualization, writing – review & editing. KJC: conceptualization, funding acquisition, project administration, supervision, visualization, writing – review & editing.

## Conflicts of interest

There are no conflicts to declare.

## Supplementary Material

AN-151-D6AN00043F-s001

## Data Availability

The data supporting this article have been included as part of the supplementary information (SI). The supporting information contains the following materials supporting the data presented in this article: a comparison of literature luminescence quantum yields for PtTFPP-based systems (Table S1); quantum yield determination for fluorous and reference nanosensors using the comparative method with Coumarin 343 (Fig. S1); rapid lifetime determination (RLD)-derived unquenched luminescence lifetimes (τ_0_) for four nanosensor formulations (Table S2); estimated radiative and non-radiative rate constants derived from quantum yield and lifetime measurements (Table S3); Stern–Volmer calibration and reversibility data for both fluorous and reference polymeric nanosensors, including hysteresis analysis (Fig. S2); residual analysis of the Stern–Volmer linear regression (Fig. S3); pH-dependent emission response (Fig. S4); 10-day luminescence stability in the presence of pyocyanin (Fig. S5); abiotic pyocyanin quenching experiments under deoxygenated (Fig. S6) and oxygenated (Fig. S7) conditions; effect of pyocyanin protonation state on reference nanosensor luminescence (Fig. S8); influence of pyocyanin on enzymatic deoxygenation kinetics (Fig. S9); hierarchical batch-to-batch variability analysis for SESE- and FNP-fabricated nanosensors with corresponding coefficient of variation tables (Fig. S10; Tables S4–S5); dynamic light scattering size distribution of fluorous nanosensors (Fig. S11); oxygenated and deoxygenated pyocyanin quenching response for four alternative nanosensor formulations (Teflon AF 2400, PVDF/PtTFPP/TFT, PVDF/PdTFPP, and PVDF/[Ru(dpp)_3_]^2+^; Fig. S12–S15); and a summary table of pyocyanin-induced quenching across all seven tested nanosensor formulations under enzymatic deoxygenation conditions (Table S6). Supplementary information is available. See DOI: https://doi.org/10.1039/d6an00043f.
