# Supplementary material for: A fluorous-phase oxygen optical nanosensor for mitigating redox-active microbial metabolite interference
Source: Analyst. 2026 Jun 15;151(15):4414–23. doi: 10.1039/d6an00043f (PMC13285658; doi:10.1039/d6an00043f)
Supplement: AN-151-D6AN00043F-s001 [file AN-151-D6AN00043F-s001.zip › fluorous nanosensor analyst supporting information v3.docx]

Supporting Information

A fluorous phase oxygen optical nanosensor for mitigating redox-active microbial metabolite interference

John M Branning, Jr.,^a,b^, Brianna M. Ruff^c^, Samuel C. Saccomano^c^, Avi I. Flamholz^d^, Dianne K. Newman^e,f,g^, Kevin J. Cash^a,c#^

1. Quantitative Biosciences and Engineering, Colorado School of Mines, Golden, CO, USA.
2. The MITRE Corporation, Bedford, Massachusetts, USA. The author’s affiliation with The MITRE Corporation is for identification purposes only and is not intended to convey or imply MITRE’s concurrence with, or support for, the positions, opinions, or viewpoints expressed by the author. Approved for Public Release, Distribution Unlimited. Public Release Case Number 24-3656.
3. Chemical and Biological Engineering Department, Colorado School of Mines, Golden, CO, USA.
4. Laboratory of Environmental Microbiology, The Rockefeller University, New York, NY 10065
5. Division of Biology and Biological Engineering, California Institute of Technology, Pasadena, California, USA
6. Resnick Sustainability Institute, California Institute of Technology, Pasadena, California, USA
7. Division of Geological and Planetary Sciences, California Institute of Technology, Pasadena, California, USA

# Address correspondence to Kevin J. Cash, [kcash@mines.edu](mailto:kcash@mines.edu)

Table S1: Literature quantum yields of PtTFPP

Figure S1: Quantum yield determination

Table S2: RLD-derived unquenched luminescence lifetimes

Table S3: Estimated photophysical rate constants for PtTFPP-based nanosensor formulations

Figure S2: Stern–Volmer reversibility

Figure S3: Stern–Volmer residual analysis

Figure S4: pH response of nanosensors

Figure S5: 10-day luminescence stability

Figure S6: Abiotic deoxygenated pyocyanin experiment

Figure S7: Abiotic oxygenated pyocyanin experiment

Figure S8: Effect of pyocyanin protonation state

Figure S9: Pyocyanin-accelerated deoxygenation

Figure S10: Variability analysis (SESE vs FNP)

Table S4: Variability analysis for SESE nanosensors

Table S5: Variability analysis for FNP nanosensors

Figure S11: DLS size distribution

Figure S12: Alternative Sensor Formulations - Teflon AF 2400 nanosensor formulation

Figure S13: Alternative Sensor Formulations - PVDF nanosensor formulation (PtTFPP)

Figure S14: Alternative Sensor Formulations - PVDF nanosensor formulation (PdTFPP)

Figure S15: Alternative Sensor Formulations - PVDF nanosensor formulation ([Ru(dpp)₃]²⁺)

Table S6: Pyocyanin-induced quenching of nanosensor formulations

**Table S1**. *Measured quantum yields (Φ) for PtTFPP-based nanosensor formulations in this work compared with representative literature values*. Reported luminescence quantum yields (Φ) of PtTFPP in molecular solutions, micellar systems, polymer matrices, and oxygen nanosensor formulations under deoxygenated or nitrogen atmospheres. Values were compiled from representative literature sources and compared with the fluorous nanosensor developed in this work. Quantum yields were determined using relative methods with appropriate reference standards as described in the cited publications. This comparison highlights the influence of microenvironment and matrix composition on the radiative efficiency of PtTFPP and contextualizes the performance of the present nanosensor system.

| System / Matrix | Environment | Quantum Yield (Φ) | Reference |
| --- | --- | --- | --- |
| Fluorous nanosensor | Deoxygenated | 0.070 | This work |
| Reference Nanosensor | Deoxygenated | 0.117 | This work |
| PtTFPP in CH₂Cl₂ | Deoxygenated | 0.088 | Zanetti, 2023 |
| PtTFPP in micelles | N₂ | 0.06–0.159 | Appl. Sci. 2019, 9, 4404^1^ |
| PtTFPP in micelles | N₂ | 0.110 | PLoS ONE 2012, 7, e33390^2^ |
| Non-FRET micelles | N₂ | 0.109 | Sens. Actuators B 2018^3^ |
| FRET micelles | N₂ | 0.231 | Sens. Actuators B 2018^3^ |
| Dual pH/O₂ nanoprobes | Deoxygenated | ~0.15 | Sens. Actuators B 2019^4^ |
| P3 micelle solution | Deoxygenated | 0.088 | Lai et al., 2004^5^ |

| 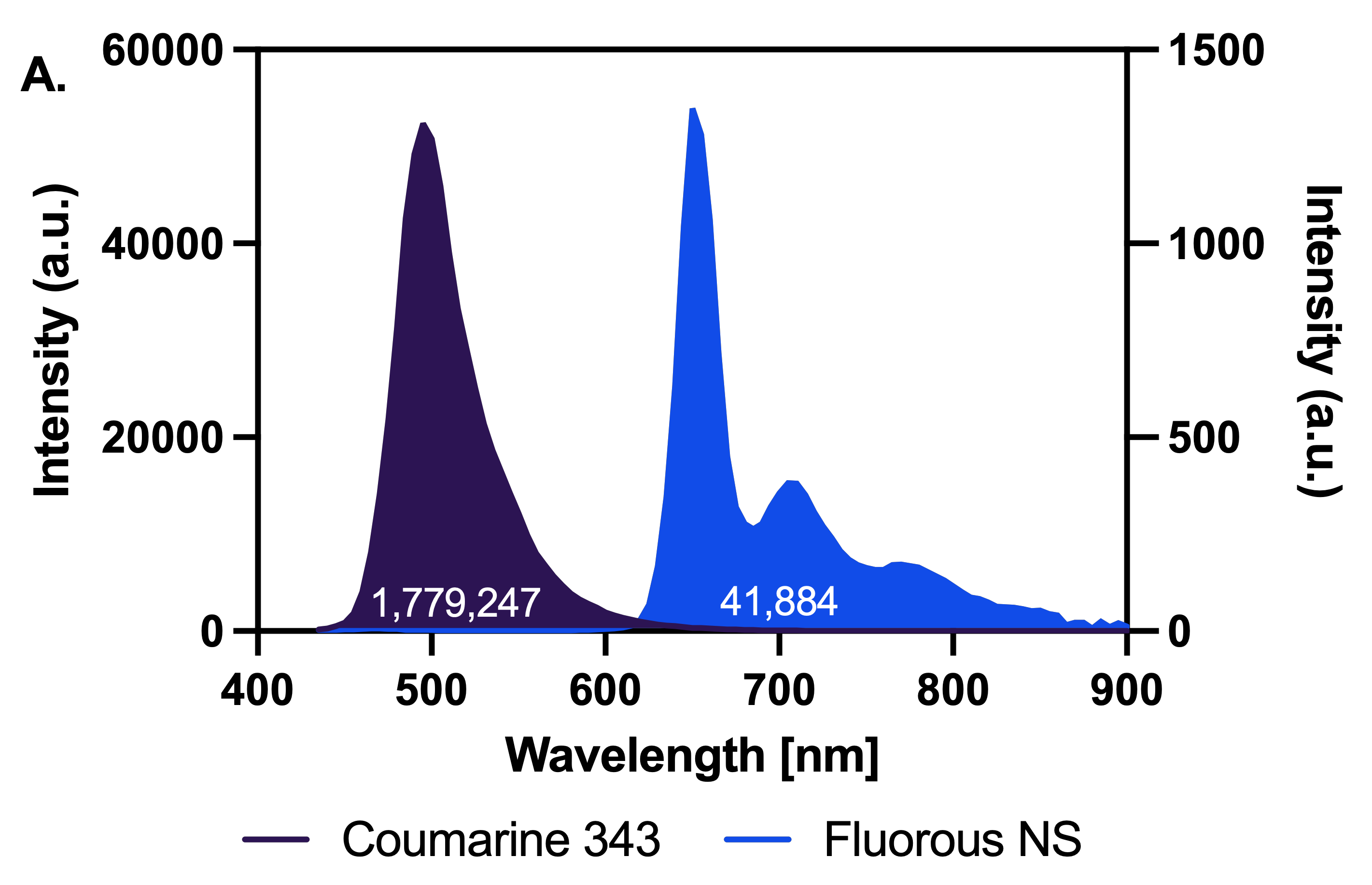 | 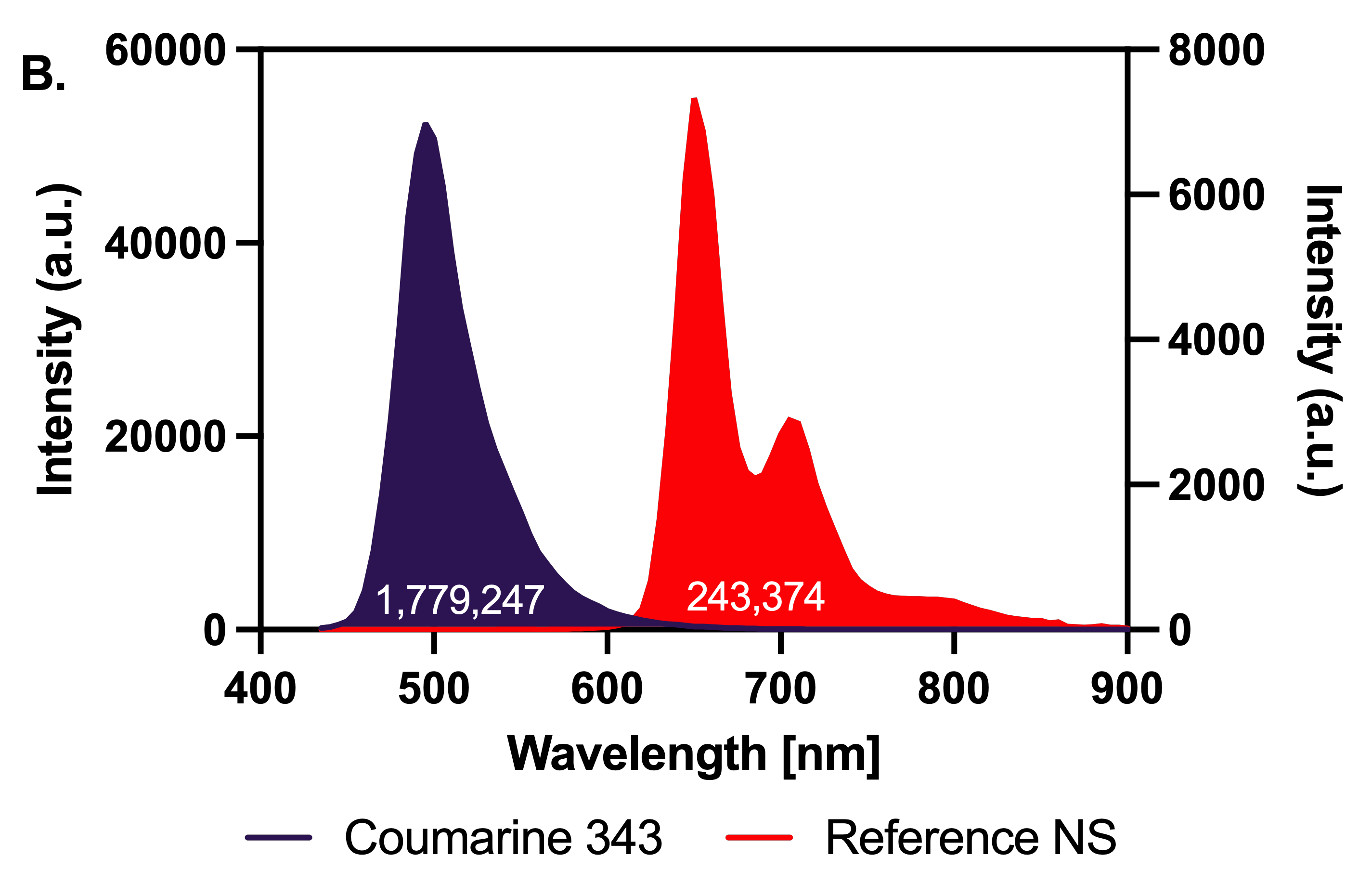 |
| --- | --- |

**Figure S1**. *Quantum yield determination*. Determination of nanosensor quantum yield using the comparative method with Coumarin 343 dissolved in ethanol as the reference fluorophore. The quantum yield (Φ) of the nanosensors (NS) was calculated using the standard comparative equation:

$$\phi_{NS}=2\phi_{Ref}\left( \frac{A_{Ref}}{A_{NS}} \right)\left( \frac{I_{NS}}{I_{Ref}} \right)\left( \frac{\eta_{NS}}{\eta_{Ref}} \right)^{2}$$

where *Φ* is the quantum yield of the dyes, *A* is the absorbance, *I* is the integrated emission intensity and η is the refractive index of the solvents (water for nanosensors, and ethanol for reference). Emission spectra were integrated from λₑₘ = 600–860 nm for both the fluorous and reference nanosensors and λₑₘ = 435–750 nm for Coumarin 343 with excitation at λₑₓ = 405 nm. The resulting quantum yield of the nanosensors was Φ_F_ = 0.070 and Φ_R_ = 0.117 at 24 °C.

**Table S2**. *RLD-derived unquenched luminescence lifetimes (τ₀) for different oxygen nanosensors formulations*. Measured under enzymatic deoxygenation conditions with λex = 405 nm, λem = 650 nm; t₁ = 20 µs. Run 1 and Run 2 are sequential technical replicates from the same nanosensor batch (n = 3 per run). Consensus τ₀ values are taken from both runs.

| NS | Matrix | Dye | $\boldsymbol{\tau}_{\mathbf{0}}\left( \boldsymbol{\mu s} \right)$ (Run 1) | $\boldsymbol{\tau}_{\mathbf{0}}\left( \boldsymbol{\mu s} \right)$ (Run 2) | Consensus $\boldsymbol{\tau}_{\mathbf{0}}\left( \boldsymbol{\mu s} \right)$ |
| --- | --- | --- | --- | --- | --- |
| NS #1 | PVC | PtTFPP | 33.7–38.3 µs | 33.3–37.9 µs | **~34–38 µs** |
| NS #2 | PVC | PtTPP | 52.1–53.5 µs | 51.9–53.2 µs | **~52–54 µs** |
| NS #3 | PVDF | PtTFPP | 62.9–69.1 µs | 62.8–69.6 µs | **~63–69 µs** |
| NS #4 | PVDF | PtTPP | 51.9–52.0 µs | 52.1–53.5 µs | **~52–54 µs** |

**Table S3**. *Estimated photophysical rate constants for PtTFPP-based nanosensor formulations*. Estimated photophysical rate constants for PtTFPP-based nanosensor formulations. The radiative rate constant ($k_{R}$) and total non-radiative rate constant ($k_{NR}$) were calculated from the measured luminescence quantum yield ($\Phi$) and unquenched phosphorescence lifetime (τ₀) using the relationships $k_{R}=\Phi/{\tau_{0}}$ and $k_{NR}=\left( 1-\Phi\right)/{\tau_{0}}$, respectively, under the assumption of purely dynamic excited-state deactivation with no unimolecular photoreaction from the triplet state.

| Formulation | $\boldsymbol{\Phi}$ | $\boldsymbol{\tau}_{\mathbf{0}}\left( \boldsymbol{\mu s} \right)$ | $\boldsymbol{k}_{\boldsymbol{R}}\left( \boldsymbol{\mu s}^{\mathbf{-1}} \right)$ | $\boldsymbol{k}_{\boldsymbol{NR}}\left( \boldsymbol{\mu s}^{\mathbf{-1}} \right)$ |
| --- | --- | --- | --- | --- |
| Reference | 0.117 | 34–38 | 0.0031–0.0034 | 0.023–0.026 |
| Fluorous | 0.070 | 63–69 | 0.0010–0.0011 | 0.013–0.015 |

| 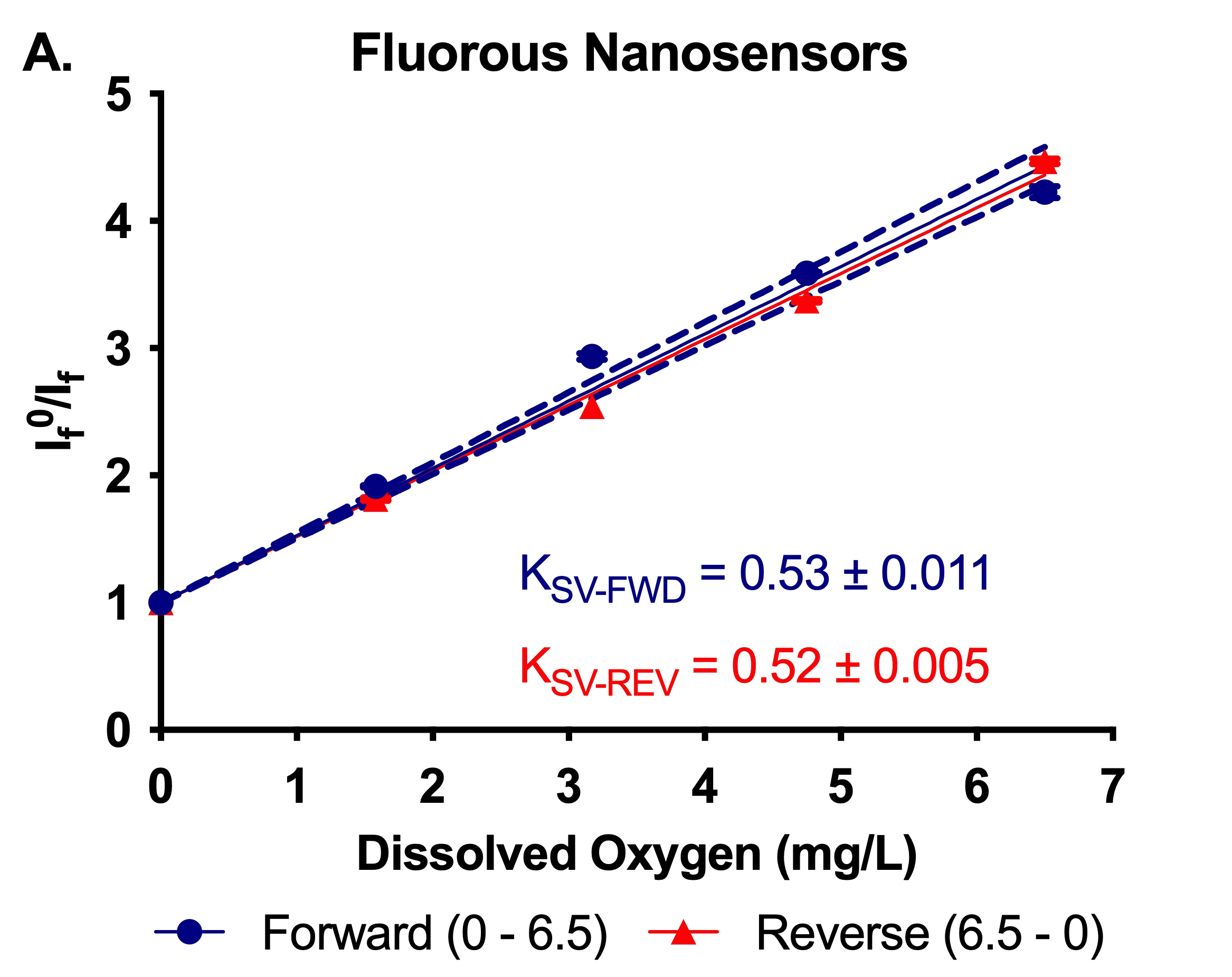 | 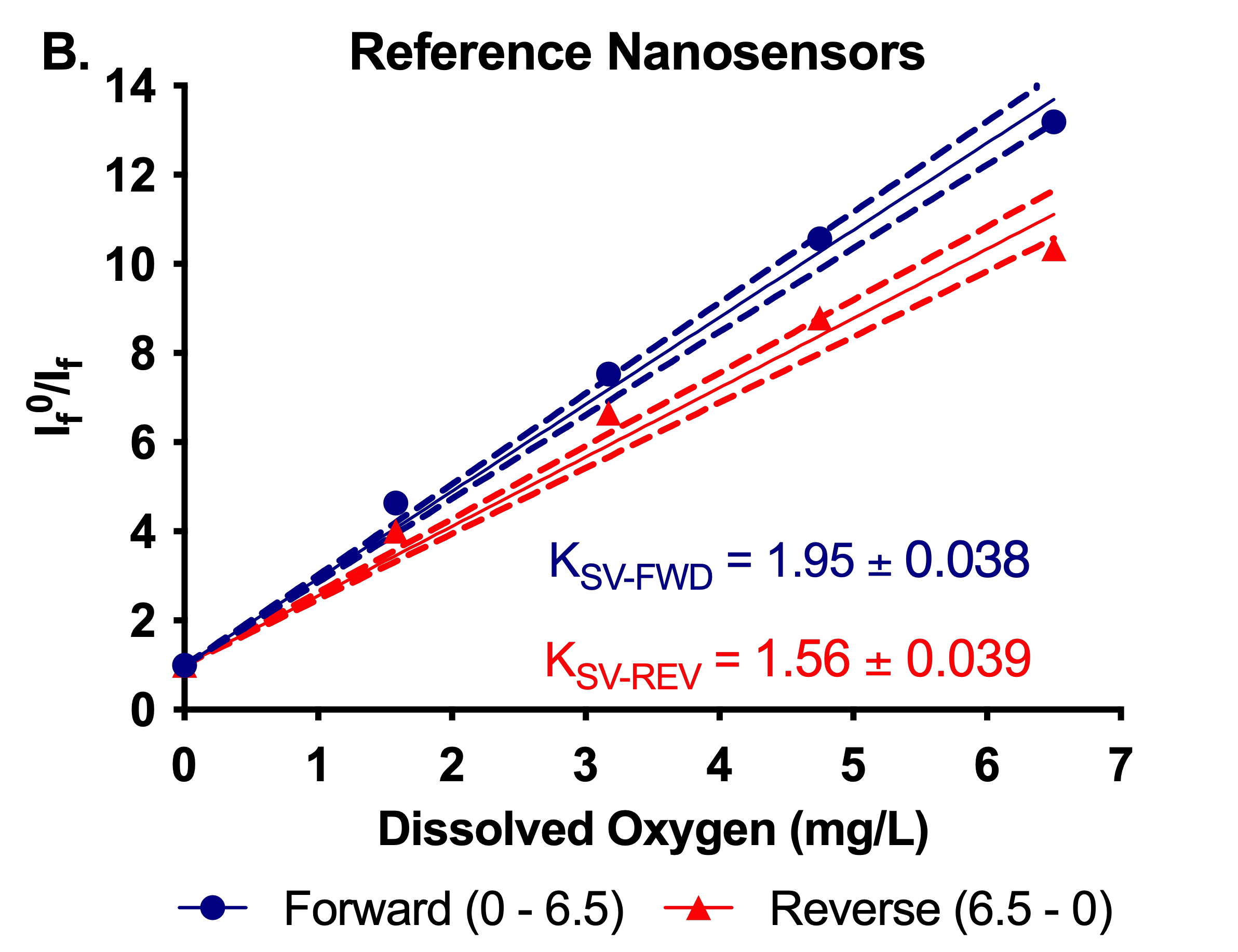 |
| --- | --- |

**Figure S2.** *Stern–Volmer calibration and reversibility of fluorous (PVDF) and reference polymeric (PVC) oxygen nanosensors.* The normalized luminescence signal is plotted as a function of dissolved oxygen concentration during sequential deoxygenation and reoxygenation cycles. The Stern–Volmer response remained linear across the tested range of 0-6.65 mg L⁻¹ dissolved O₂. Error bars represent the standard deviation at each oxygen concentration (n = 3), and the dotted lines represent the 95% confidence interval of the linear regression. (A) Fluorous nanosensors (PVDF matrix, PFPE-PEG-5K surfactant): K_SV-FWD_ = 0.53 ± 0.011 L mg⁻¹, K_SV-REV_ = 0.52 ± 0.005 L mg⁻¹. (B) Reference polymeric nanosensors (PVC matrix, PEG-750 surfactant): K_SV-FWD_ = 1.95 ± 0.038 L mg⁻¹, K_SV-REV_ = 1.56 ± 0.039 L mg⁻¹. The approximately three-fold difference in K_SV_ between the two systems reflects differences in matrix composition and oxygen partitioning behavior. The reference nanosensors exhibited notable hysteresis (Figure S2B) between forward and reverse KSV values, whereas the fluorous nanosensors showed near-perfect forward/reverse agreement.

**Figure S3.** *Residual analysis of the Stern–Volmer regression for fluorous nanosensors.* Deviations (Δ) from the linear regression fit are plotted across the measured dissolved oxygen concentration range. Residuals exhibit systematic curvature characterized by positive deviations at low to intermediate O₂ concentrations and negative deviations at the highest O₂ levels, indicating departure from ideal linear Stern–Volmer quenching behavior.

**Figure S4**. *pH-dependent emission response of fluorous nanosensors measured at atmospheric oxygen (6.65 mg L⁻¹ O₂)*. Measurements were conducted across the pH range 3.0-9.5. The mean luminescence intensity across all measurements was 3354 ± 141 a.u. (n = 3). One-way ANOVA indicated a statistically significant difference across pH conditions (F = 10.28, p = 0.00019). Tukey’s post hoc analysis showed that the statistical difference was primarily driven by elevated luminescence at pH 6.0 (~12% above the grand mean), while the remaining pH conditions varied within approximately ±5% of the mean without a monotonic pH-dependent trend.

**Figure S5.** *Normalized luminescence stability of nanosensors over a 10-day period.* Signal intensity on day 1 was normalized to 0% decay, and values represent the percent decrease in luminescence relative to the initial signal. Reference polymeric nanosensors exhibited a 16.8% decrease in intensity over 10 days, whereas fluorous-phase nanosensors showed only a 1.3% decrease while maintaining higher residual luminescence levels.

**Figure S6.** *Abiotic deoxygenated pyocyanin experiment.* Normalized deoxygenated luminescence response of fluorous and traditional polymeric nanosensors in the presence and absence of pyocyanin under abiotic deoxygenation conditions. Nanosensor solutions were deoxygenated by nitrogen bubbling (20 mL min⁻¹) for 20 min to establish baseline luminescence (λₑₓ = 405 nm). Subsequently, 100 μL of deoxygenated pyocyanin in PBS was injected while nitrogen bubbling continued. Luminescence measurements were collected after an additional 10 min of bubbling.

**Figure S7.** *Abiotic oxygenated pyocyanin experiment.* Normalized oxygenated luminescence response of fluorous and traditional polymeric nanosensors in the presence of varying concentrations of pyocyanin under abiotic oxygenation conditions. Nanosensor solutions were oxygenated by air bubbling (20 mL min⁻¹) for 20 min to establish baseline luminescence (λₑₓ = 405 nm). Subsequently, 100 μL of oxygenated pyocyanin in PBS was injected while air bubbling continued. Quenching measurements were recorded after 10 min of bubbling.

|  |  |
| --- | --- |

**Figure S8.** *Effect of pyocyanin protonation state on quenching of traditional polymeric oxygen nanosensors.* (A) Normalized luminescence spectra before and after pyocyanin injection. (B) Normalized endpoint luminescence intensity before and after pyocyanin injection. Nanosensor solutions were deoxygenated by nitrogen bubbling (20 mL min⁻¹) for 20 min to establish baseline luminescence (λₑₓ = 405 nm). Subsequently, 500 μL of deoxygenated pyocyanin prepared in Britton–Robinson buffer was injected while nitrogen bubbling continued. Measurements were collected after an additional 10 min of bubbling.

**Figure S9.** *Influence of pyocyanin on enzymatic deoxygenation kinetics*. In the absence of pyocyanin, oxygen concentration decreased gradually during the glucose/glucose oxidase reaction. In the presence of pyocyanin, the time required to reach near-zero dissolved oxygen was reduced by more than 200 s. Dissolved oxygen concentrations were measured using a microsensor electrode (Unisense, Aarhus, Denmark).

| 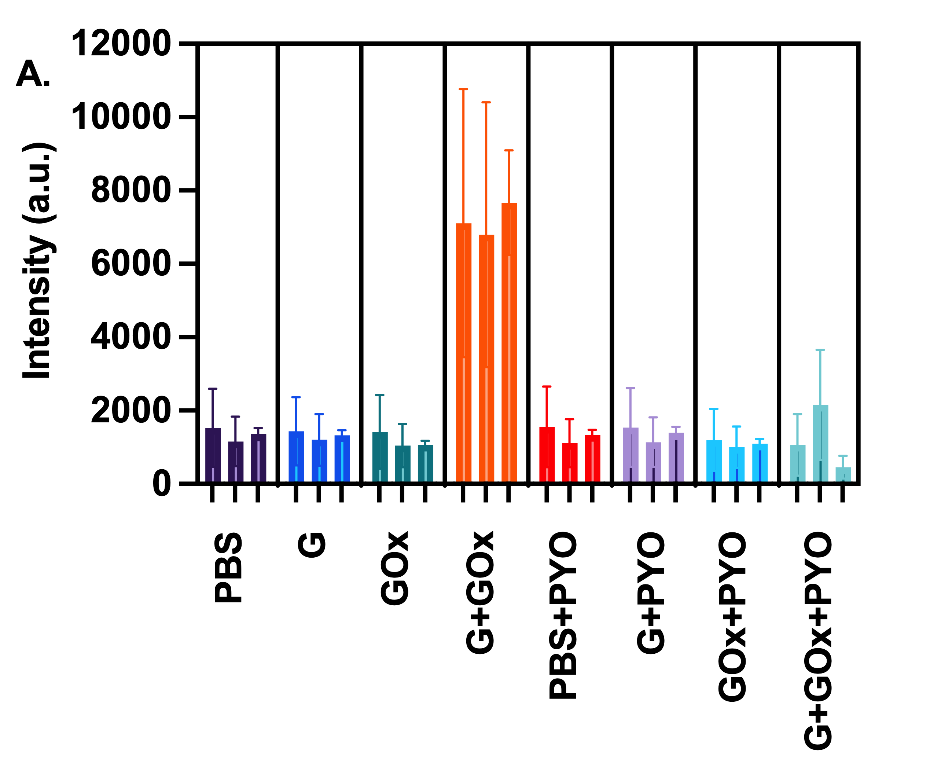 | 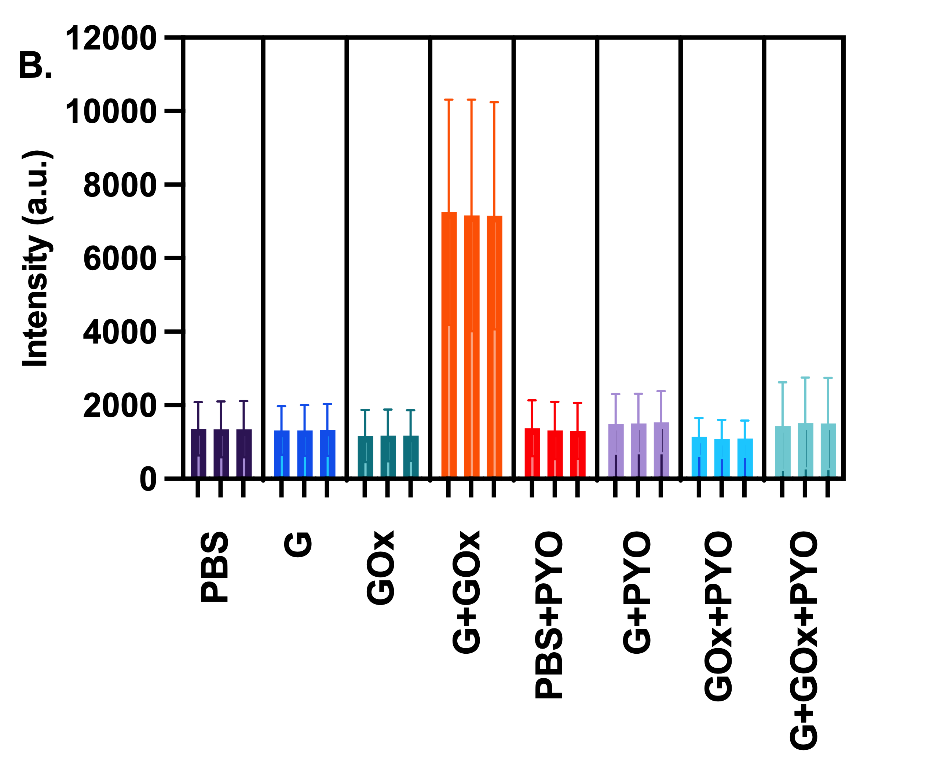 |
| --- | --- |
| 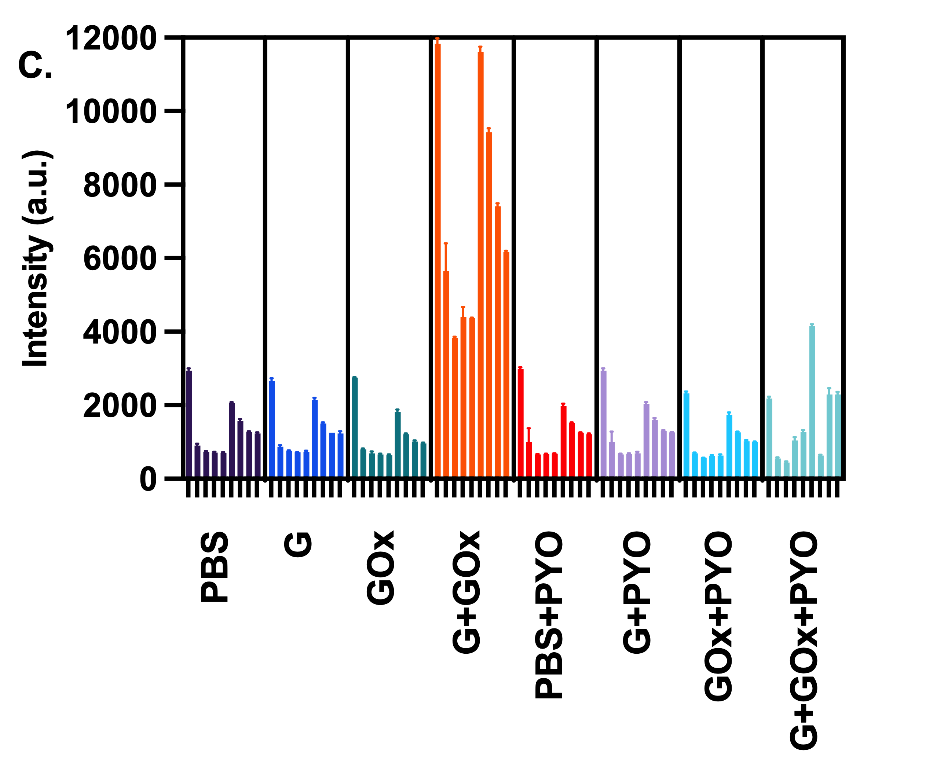 | 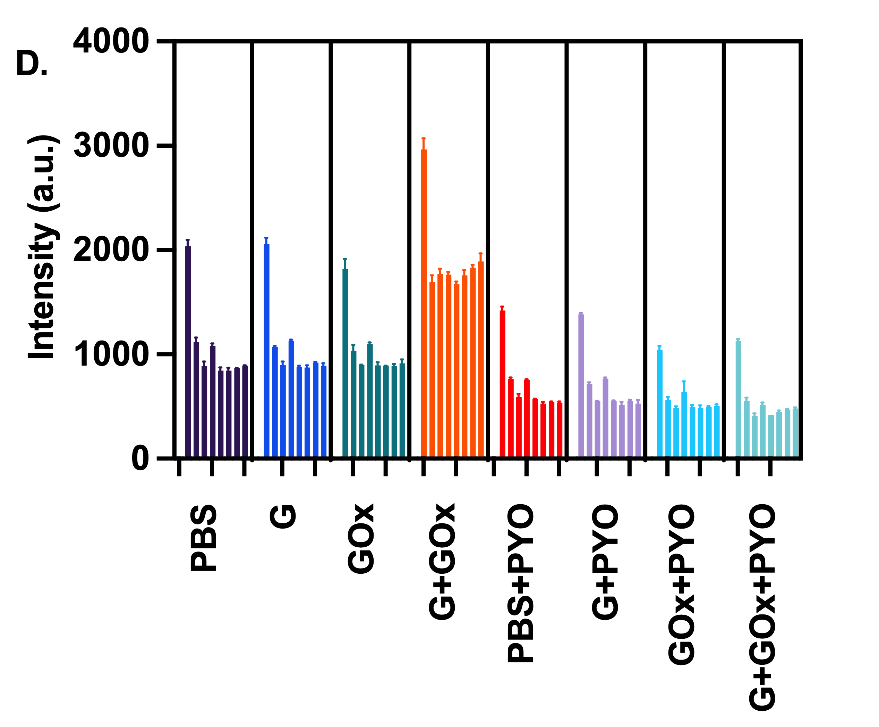 |

**Figure S10**. *Hierarchical variability analysis of fluorous nanosensor response fabricated using solvent exchange self-emulsification (SESE) (A–C) and flash nanoprecipitation (FNP) (D).* Variability is shown across independently prepared batches and measurement replicates. (A) Variability grouped by optode batch (inter-optode variability). (B) Total measurement variability across optodes and nanosensor solutions. (C) Variability across nanosensor solutions derived from each optode (intra-optode variability). (D) Corresponding variability analysis for FNP-fabricated nanosensors.

**Table S4.** *Coefficient of variation (CV%) for SESE-fabricated fluorous nanosensors under each experimental condition.* Variability is reported at three hierarchical levels: (i) overall condition variability, (ii) variability across independently prepared optode batches (inter-optode variability), and (iii) variability across nanosensor solutions derived from each optode (intra-optode variability). Within-solution CV values are reported as mean ± SD of replicate measurements (n = 9).

| Condition | Overall CV (%) | CV Across Optode Batches (%) | CV Across Nanosensor Solutions (%) |
| --- | --- | --- | --- |
| PBS | 52.5 | 65.8 / 54.7 / 11.5 | 2 / 6 / 2.9 / 2.7 / 2.6 / 1.3 / 3.1 / 1.7 / 2.2 |
| G | 49.0 | 61.1 / 55.6 / 9.5 | 2.5 / 4.8 / 2 / 1.1 / 3.2 / 3 / 2 / 0.2 / 4.9 |
| GOx | 56.3 | 66.8 / 53.2 / 10.5 | 0.3 / 3.3 / 7.3 / 3.4 / 3.5 / 3.3 / 2.2 / 3.3 / 2.6 |
| G + GOx | 40.7 | 48.5 / 50.2 / 17.5 | 1.3 / 13.3 / 0.7 / 6.1 / 0.3 / 1.3 / 1.1 / 1.1 / 0.6 |
| PBS + PYO | 54.4 | 66.9 / 55.5 / 10.3 | 1.5 / 37 / 2.1 / 2 / 2.3 / 3.2 / 0.8 / 0.4 / 1.4 |
| G + PYO | 52.7 | 65.6 / 56 / 11.2 | 2.1 / 27.5 / 2.2 / 3.4 / 4.7 / 2.4 / 2.5 / 1.7 / 1.2 |
| GOx + PYO | 51.2 | 66.7 / 52.8 / 10.9 | 2.2 / 3.1 / 1.3 / 3.6 / 5.5 / 3.2 / 1.6 / 2.6 / 1.4 |
| G + GOx + PYO | 68.6 | 74.6 / 65.6 / 45.2 | 2.6 / 4.1 / 5.5 / 9.2 / 4 / 1.3 / 3.1 / 7.5 / 3 |

**Table S5.** *Coefficient of variation (CV%) for FNP-fabricated fluorous nanosensors under each experimental condition.* Variability is reported at three hierarchical levels: (i) overall condition variability, (ii) variability across independently prepared optode batches (inter-optode variability), and (iii) variability across nanosensor solutions derived from each optode (intra-optode variability). Within-solution CV values are reported as mean ± SD of replicate measurements (n = 9).

| Condition | Overall CV (%) | CV Across Optode Batches (%) | CV Across Nanosensor Solutions (%) |
| --- | --- | --- | --- |
| PBS | 34.7 | 23.9 / 11.4 / 2.5 | 3.1 / 2.9 / 3.7 / 5.1 / 2 / 3.4 / 3 / 0.2 / 0.6 |
| G | 33.8 | 25.8 / 12 / 2.7 | 2.1 / 2.9 / 0.9 / 3.7 / 0.8 / 1.6 / 2.3 / 0.9 / 2.7 |
| GOx | 28.1 | 22.8 / 10.1 / 2.7 | 3.8 / 5.1 / 5.4 / 0.4 / 1.3 / 3.3 / 0.3 / 2.6 / 4.1 |
| G + GOx | 20.4 | 22.5 / 3.1 / 3.8 | 3.3 / 3.6 / 3.8 / 2.7 / 1.5 / 1.5 / 2.8 / 1.3 / 4 |
| PBS + PYO | 38.6 | 24.6 / 13.2 / 2.1 | 1.7 / 2.6 / 1.8 / 5.5 / 0.7 / 0.4 / 3 / 0.8 / 1.9 |
| G + PYO | 38.3 | 25.9 / 16.3 / 5.1 | 0.1 / 0.9 / 1.7 / 1 / 1.4 / 0.8 / 5.7 / 1.7 / 6.3 |
| GOx + PYO | 30.2 | 24.5 / 16 / 3.6 | 1.5 / 3.4 / 4.9 / 3.4 / 16 / 4.2 / 5.7 / 2 / 2.9 |
| G + GOx + PYO | 39.1 | 27.9 / 11.3 / 3.2 | 2.5 / 1.4 / 4.9 / 5.8 / 4.1 / 0.3 / 2.6 / 1.1 / 2 |


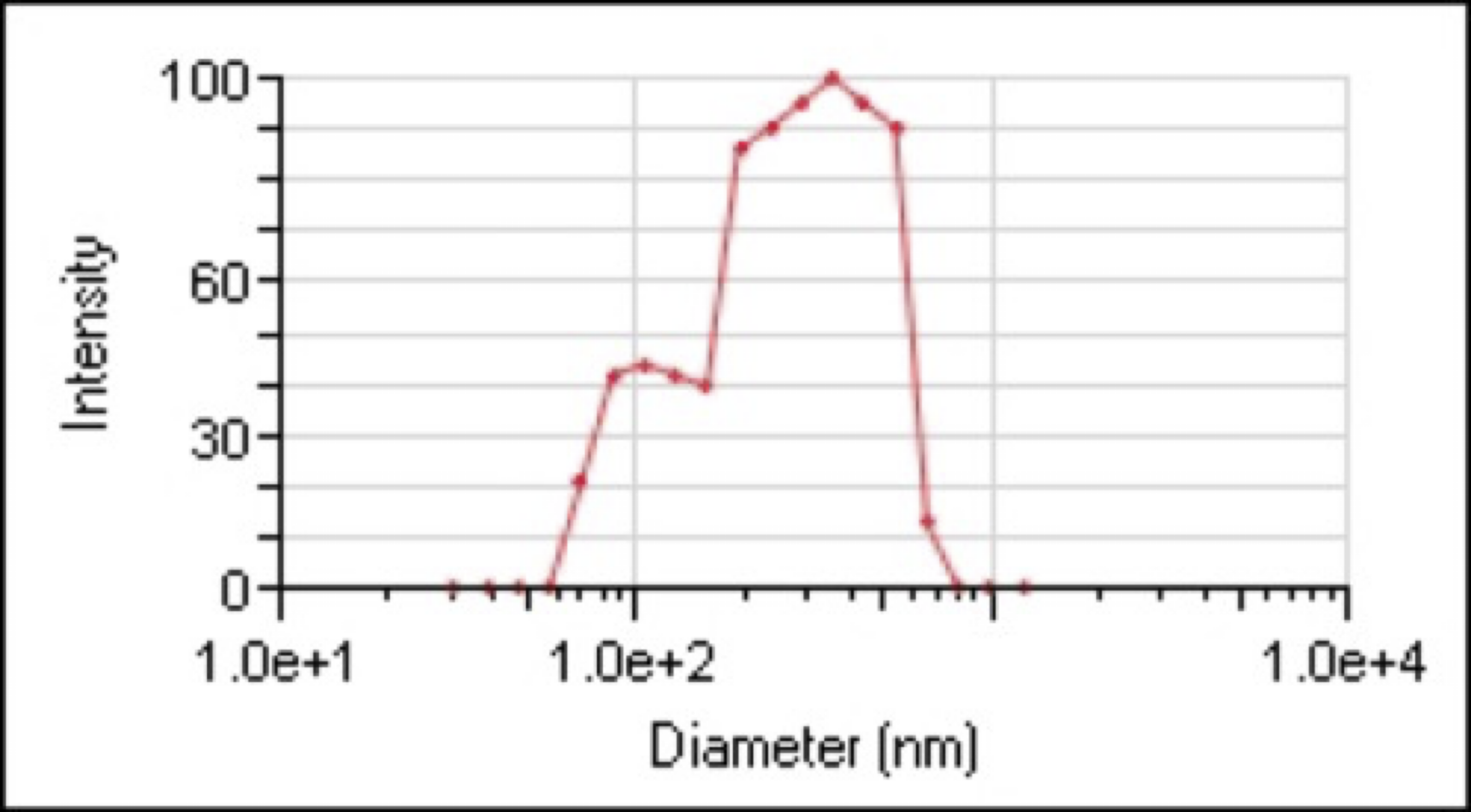


**Figure S11.** *Dynamic light scattering (DLS) analysis of fluorous nanosensor size distribution*. The nanosensors exhibited an average hydrodynamic diameter of 212 nm with a polydispersity index of 0.27 (n = 3).


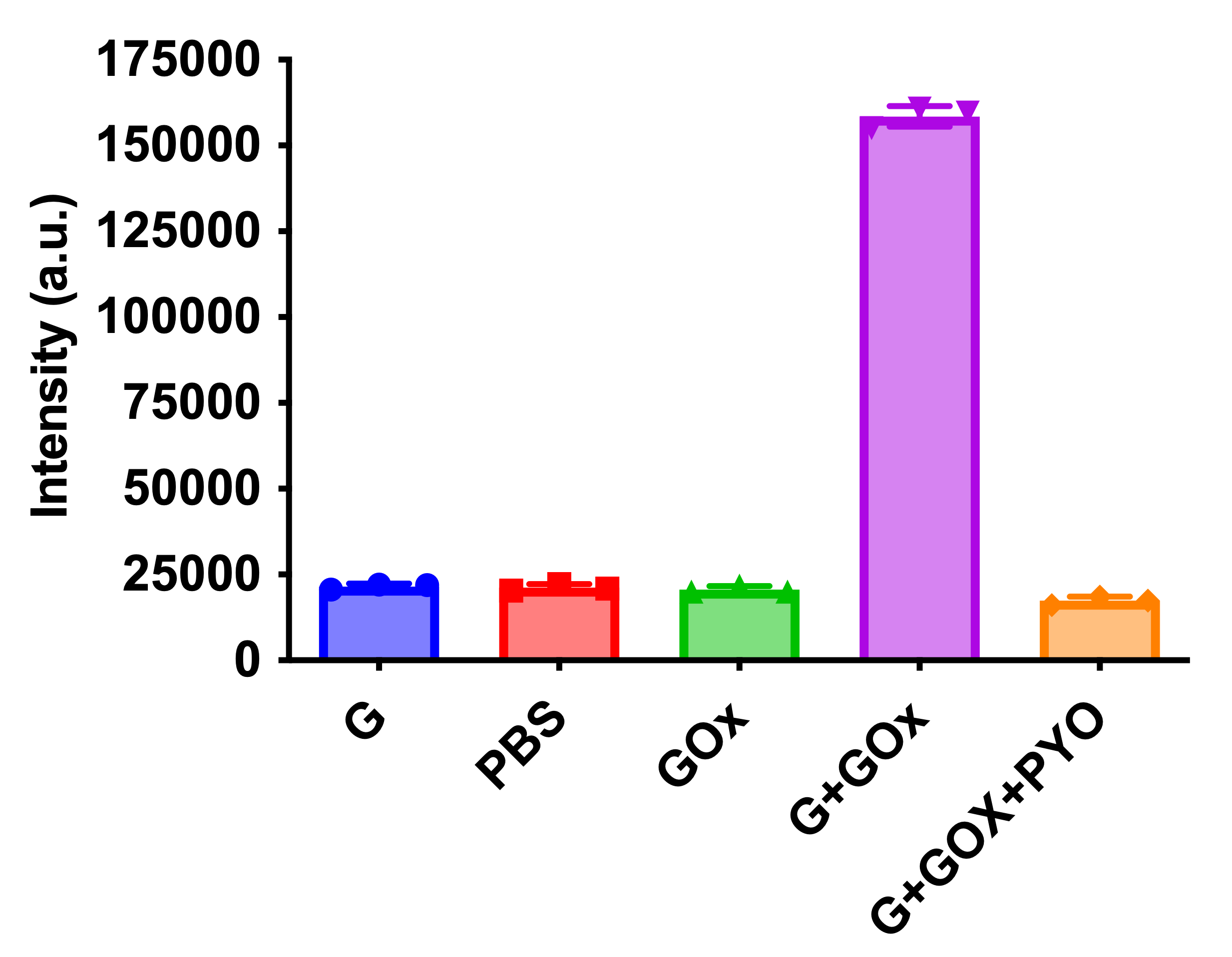


**Figure S12.** *Alternative Sensor Formulations - Teflon AF 2400 nanosensor formulation*. Oxygenated and deoxygenated response of fluorous oxygen-sensitive nanosensors fabricated using Teflon AF 2400 polymer, PtTFPP dye, hexafluorobenzene solvent, and PFPE-PEG-5K surfactant. Although the nanosensors retained oxygen sensitivity, no improvement in resistance to pyocyanin-induced quenching was observed relative to the reference nanosensors.

**Figure S13.** *Alternative Sensor Formulations - PVDF nanosensor formulation (PtTFPP).* Oxygenated and deoxygenated response of fluorous oxygen-sensitive nanosensors fabricated using PVDF polymer, PtTFPP dye, trifluorotoluene solvent, and PFPE-PEG-5K surfactant. While the nanosensors maintained oxygen sensitivity, pyocyanin rejection was not improved relative to the reference nanosensors.

**Figure S14.** *Alternative Sensor Formulations - PVDF nanosensor formulation (PdTFPP).* Oxygenated and deoxygenated response of fluorous oxygen-sensitive nanosensors fabricated using PVDF polymer, PdTFPP dye, trifluorotoluene solvent, and PFPE-PEG-5K surfactant. No improvement in resistance to pyocyanin-induced quenching was observed compared with the reference nanosensors.

**Figure S15.** *Alternative Sensor Formulations - PVDF nanosensor formulation ([Ru(dpp)₃]²⁺).* Oxygenated and deoxygenated response of fluorous oxygen-sensitive nanosensors fabricated using PVDF polymer, [Ru(dpp)₃]²⁺ dye, THF solvent, and PFPE-PEG-5K surfactant. These nanosensors exhibited greater sensitivity to pyocyanin-induced quenching relative to both the reference and fluorous nanosensor systems.

**Table S6.** *Pyocyanin-induced quenching of nanosensor formulations under enzymatic deoxygenation conditions*. Luminescence intensities were measured before and after addition of pyocyanin (PYO), and the remaining luminescence and percent quenching were calculated relative to the deoxygenated baseline. Nanosensor formulations incorporating fluorous polymer matrices with PtTFPP exhibited substantially improved resistance to pyocyanin-induced quenching compared with conventional PVC-based reference formulations, despite lower intrinsic oxygen sensitivity. The PVDF+PtTFPP (primary) formulation demonstrated the greatest pyocyanin resistance among the tested fluorous oxygen-sensitive formulations, retaining 47.8% of its initial deoxygenated luminescence following pyocyanin exposure. Values reported here were obtained from an independent experimental run using separately prepared nanosensor batches from those used in Figure 4, and differences in absolute quenching percentages between these datasets are consistent with the batch-to-batch variability documented in Figure S10 and Tables S4–S5.

| Polymer | Dye Fluorinated | G + Gox Mean  Intensity (a.u.) | G + Gox + PYO  Mean Intensity (a.u.) | Luminescence Remaining (%) | Quenching (%) |
| --- | --- | --- | --- | --- | --- |
| PVC | PtTFPP | 13221 | 34244 | 25.9 | 74.1 |
| PVC | PtTpp | 38587 | 4945 | 12.8 | 87.2 |
| PVDF | PtTFPP | 5714 | 2730 | 47.8 | 52.2 |
| PVDF | PtTPP | 354 | 228 | 64.6 | 35.4**^††^** |
| PVDF | PdTFPP | 149013 | 9970 | 6.7 | 93.3 |
| PVDF | [Ru(dpp)₃]²⁺ | 18098 | 3170 | 17.5 | 82.5 |
| PVDF | PtTFPP**^†^** | 53333 | 14085 | 26.4 | 73.6 |
| Teflon | PtTFPP | 158505 | 17405 | 11.0 | 89.0 |

**^†^Note: Solvent** α,α,α-Trifluorotoluene (TFT)

**^††^Note:** PVDF+PtTPP exhibited the lowest pyocyanin-induced quenching among all formulations tested (35.4%). However, its oxygenated (137 ± 12 a.u.) and deoxygenated (354 ± 22 a.u.) baseline signal intensities were substantially lower than those of the other formulations, limiting its utility for biological imaging and sensing applications. Consequently, this formulation was excluded from primary performance comparisons but is reported for completeness and as a potential avenue for future optimization.

(1) Zanetti, C.; Larkin, E.; Maguire, A. R.; Papkovsky, D. B. Hetero-Substituted Derivative of PtPFPP for Intracellular O2 Sensing of Mammalian Cells. In *Optical Sensors 2023*; Lieberman, R. A., Baldini, F., Homola, J., Eds.; SPIE: Prague, Czech Republic, 2023; p 73. https://doi.org/10.1117/12.2669180.

(2) Su, F.; Alam, R.; Mei, Q.; Tian, Y.; Youngbull, C.; Johnson, R. H.; Meldrum, D. R. Nanostructured Oxygen Sensor - Using Micelles to Incorporate a Hydrophobic Platinum Porphyrin. *PLoS ONE* **2012**, *7* (3), e33390. https://doi.org/10.1371/journal.pone.0033390.

(3) Zhao, Q.; Pan, T.; Xiang, G.; Mei, Z.; Jiang, J.; Li, G.; Zou, X.; Chen, M.; Sun, D.; Jiang, S.; Tian, Y. Highly Efficient Ratiometric Extracellular Oxygen Sensors through Physical Incorporation of a Conjugated Polymer and PtTFPP in Graft Copolymers. *Sensors and Actuators B: Chemical* **2018**, *273*, 242–252. https://doi.org/10.1016/j.snb.2018.06.026.

(4) Pan, T.; Yang, C.; Shi, J.; Hao, C.; Qiao, Y.; Li, J.; Deng, M.; Tian, Y.; Chen, M. Dual pH and Oxygen Luminescent Nanoprobes Based on Graft Polymers for Extracellular Metabolism Monitoring and Intracellular Imaging. *Sensors and Actuators B: Chemical* **2019**, *291*, 306–318. https://doi.org/10.1016/j.snb.2019.04.082.

(5) Lai, S.-W.; Hou, Y.-J.; Che, C.-M.; Pang, H.-L.; Wong, K.-Y.; Chang, C. K.; Zhu, N. Electronic Spectroscopy, Photophysical Properties, and Emission Quenching Studies of an Oxidatively Robust Perfluorinated Platinum Porphyrin. *Inorg. Chem.* **2004**, *43* (12), 3724–3732. https://doi.org/10.1021/ic049902h
