# Supplementary figures and images for: A fluorous-phase oxygen optical nanosensor for mitigating redox-active microbial metabolite interference
Source: Analyst. 2026 Jun 15;151(15):4414–23. doi: 10.1039/d6an00043f (PMC13285658; doi:10.1039/d6an00043f)

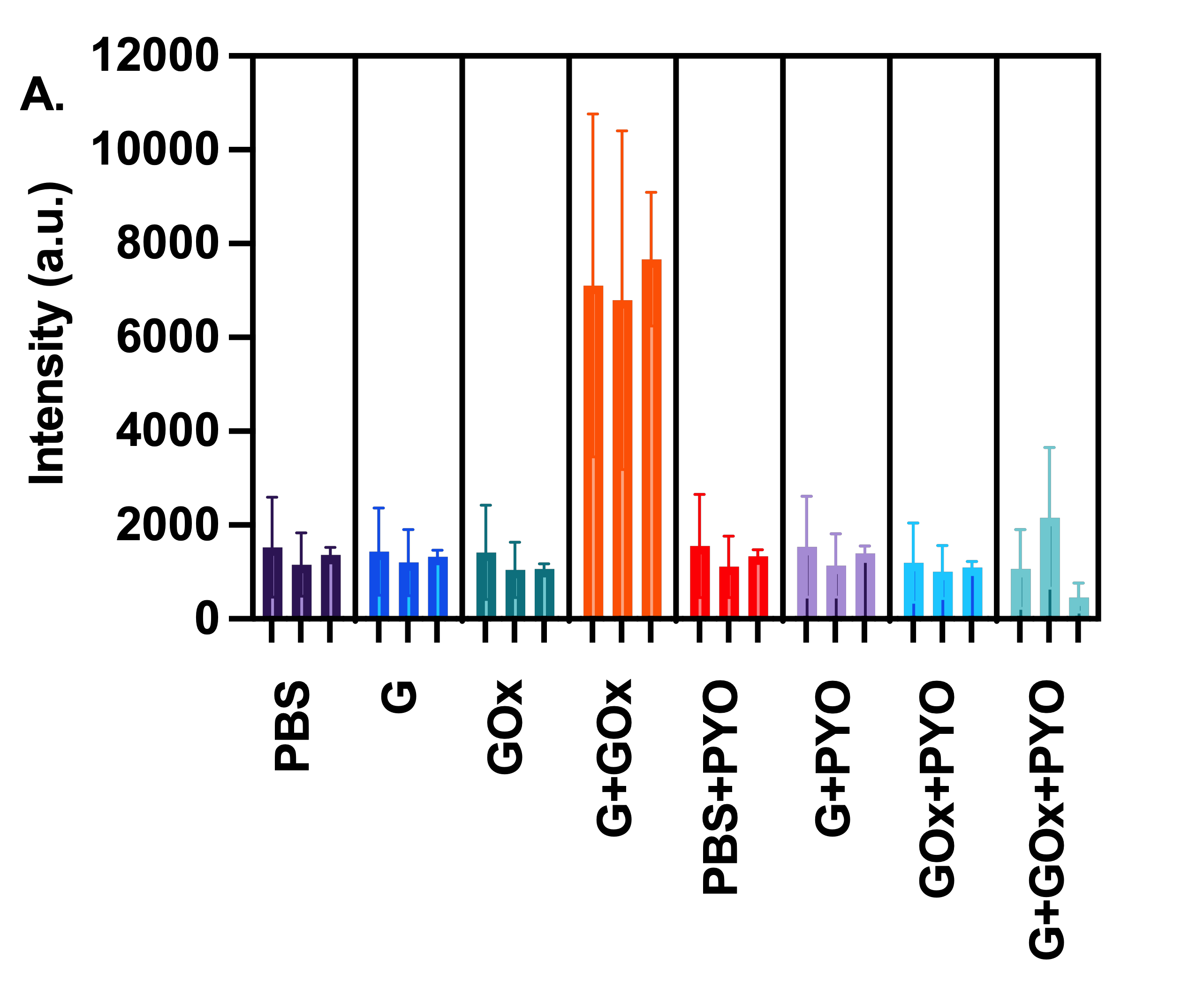

Supplement: AN-151-D6AN00043F-s001 [file AN-151-D6AN00043F-s001.zip › manuscript figure s10a.tiff]

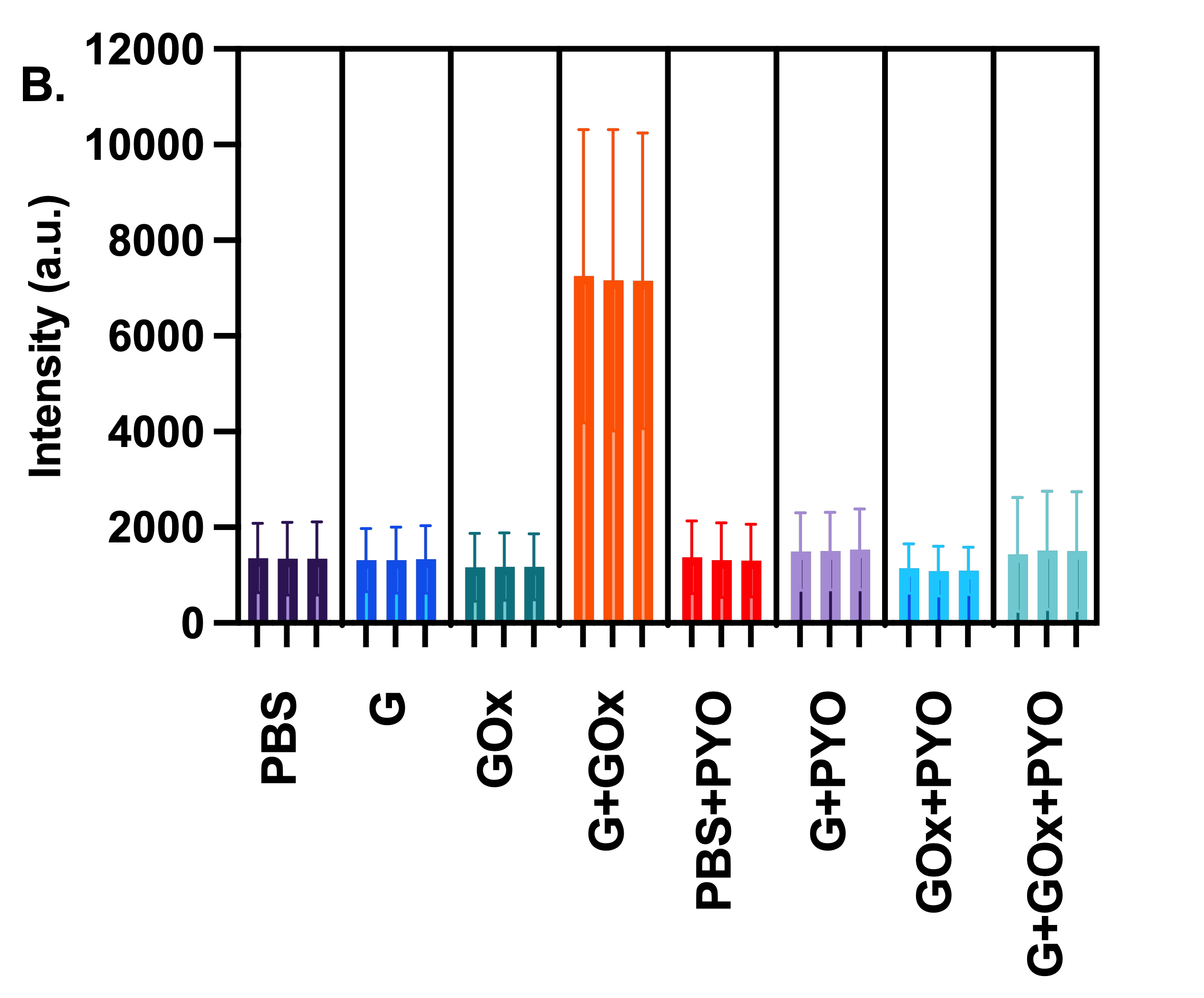

Supplement: AN-151-D6AN00043F-s001 [file AN-151-D6AN00043F-s001.zip › manuscript figure s10b.tiff]

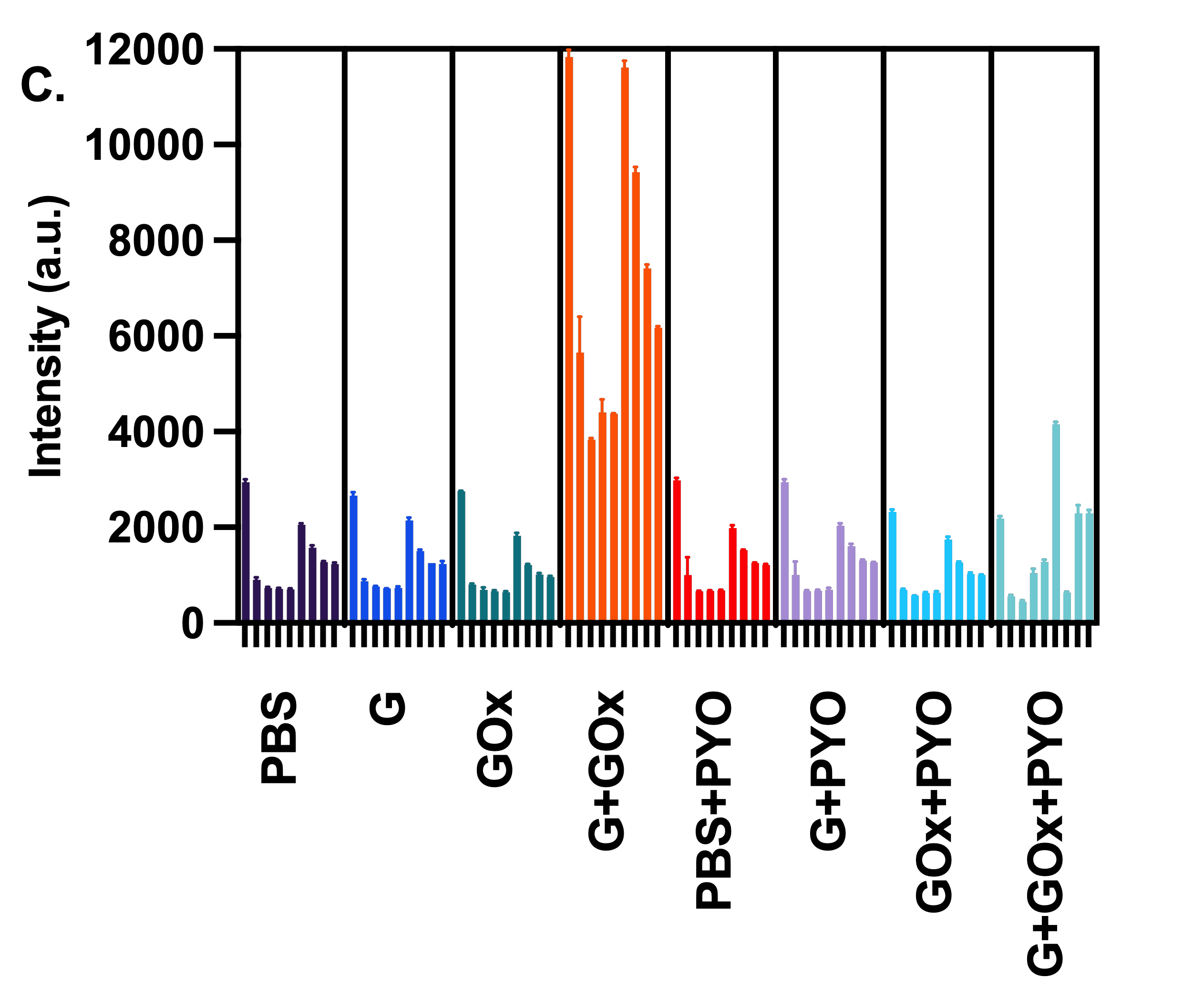

Supplement: AN-151-D6AN00043F-s001 [file AN-151-D6AN00043F-s001.zip › manuscript figure s10c.tiff]

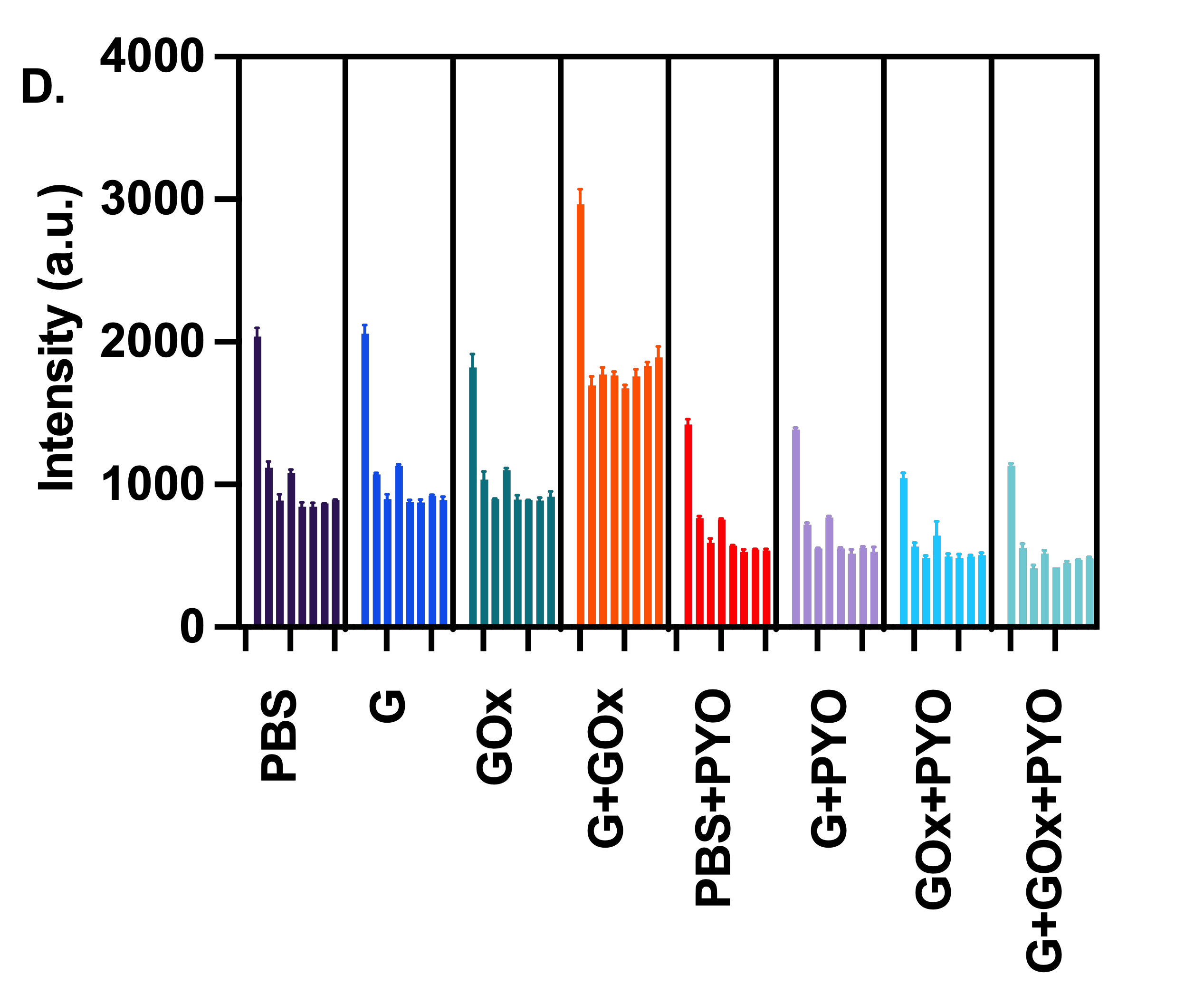

Supplement: AN-151-D6AN00043F-s001 [file AN-151-D6AN00043F-s001.zip › manuscript figure s10d.tiff]

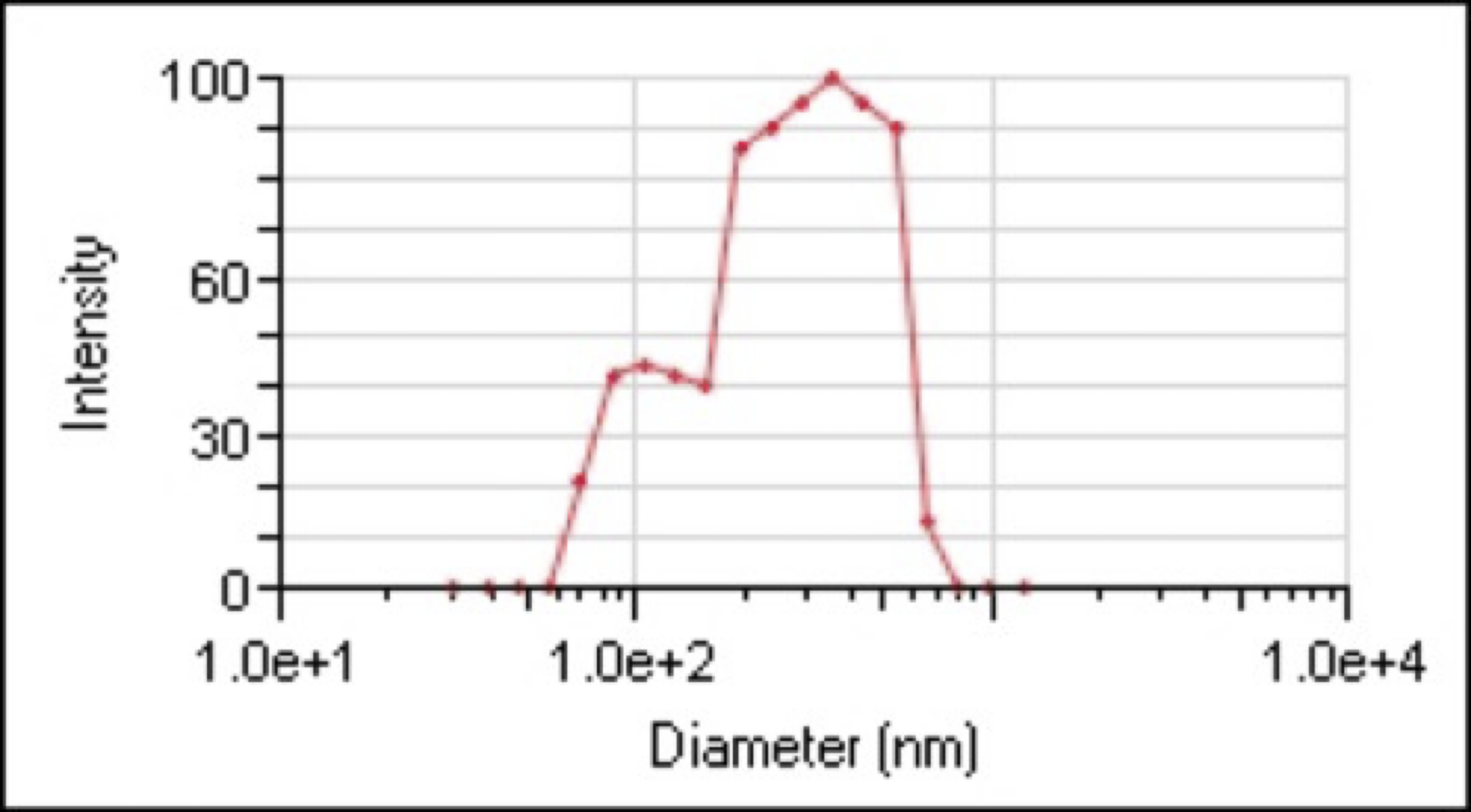

Supplement: AN-151-D6AN00043F-s001 [file AN-151-D6AN00043F-s001.zip › manuscript figure s11.tiff]

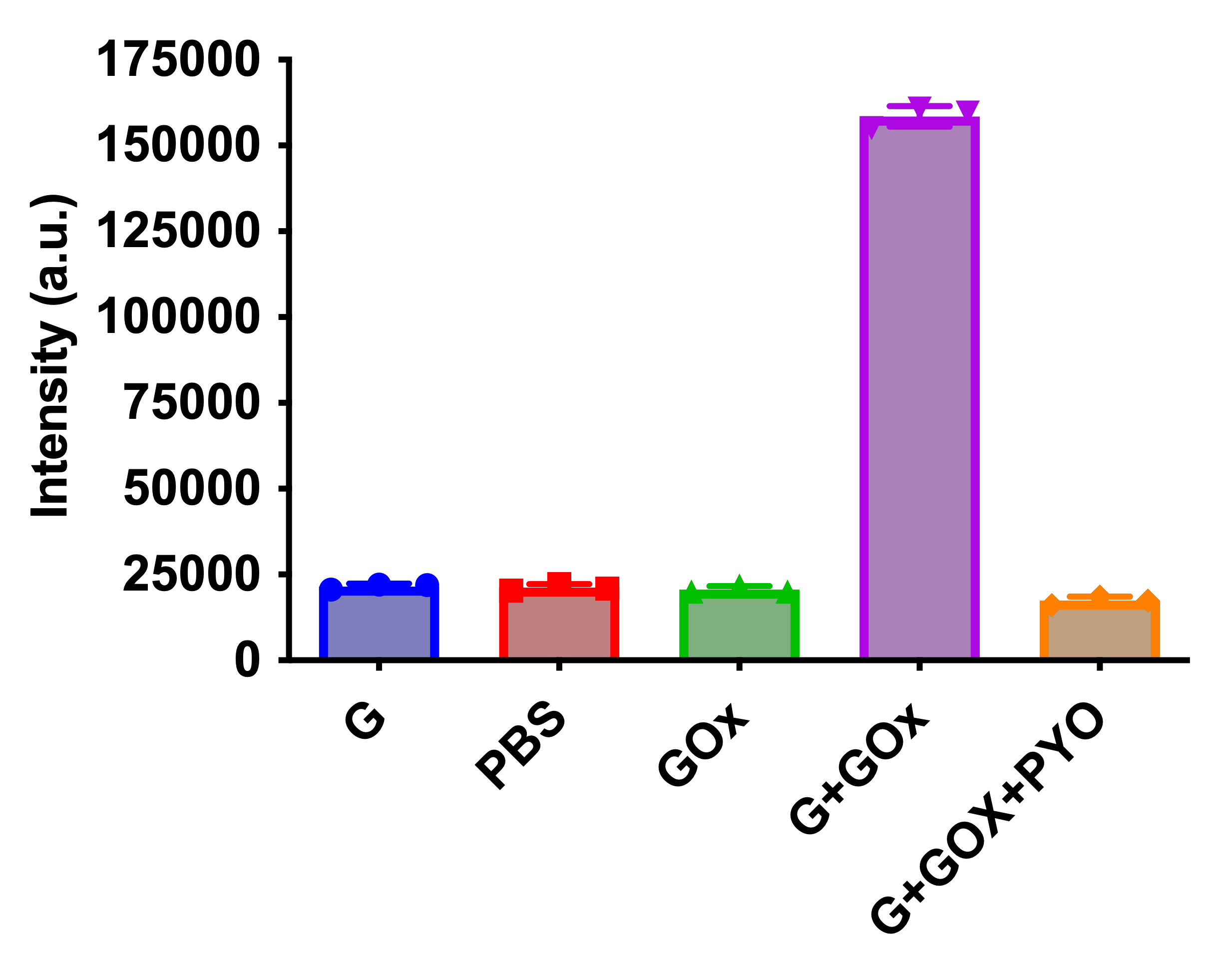

Supplement: AN-151-D6AN00043F-s001 [file AN-151-D6AN00043F-s001.zip › manuscript figure s12.tiff]

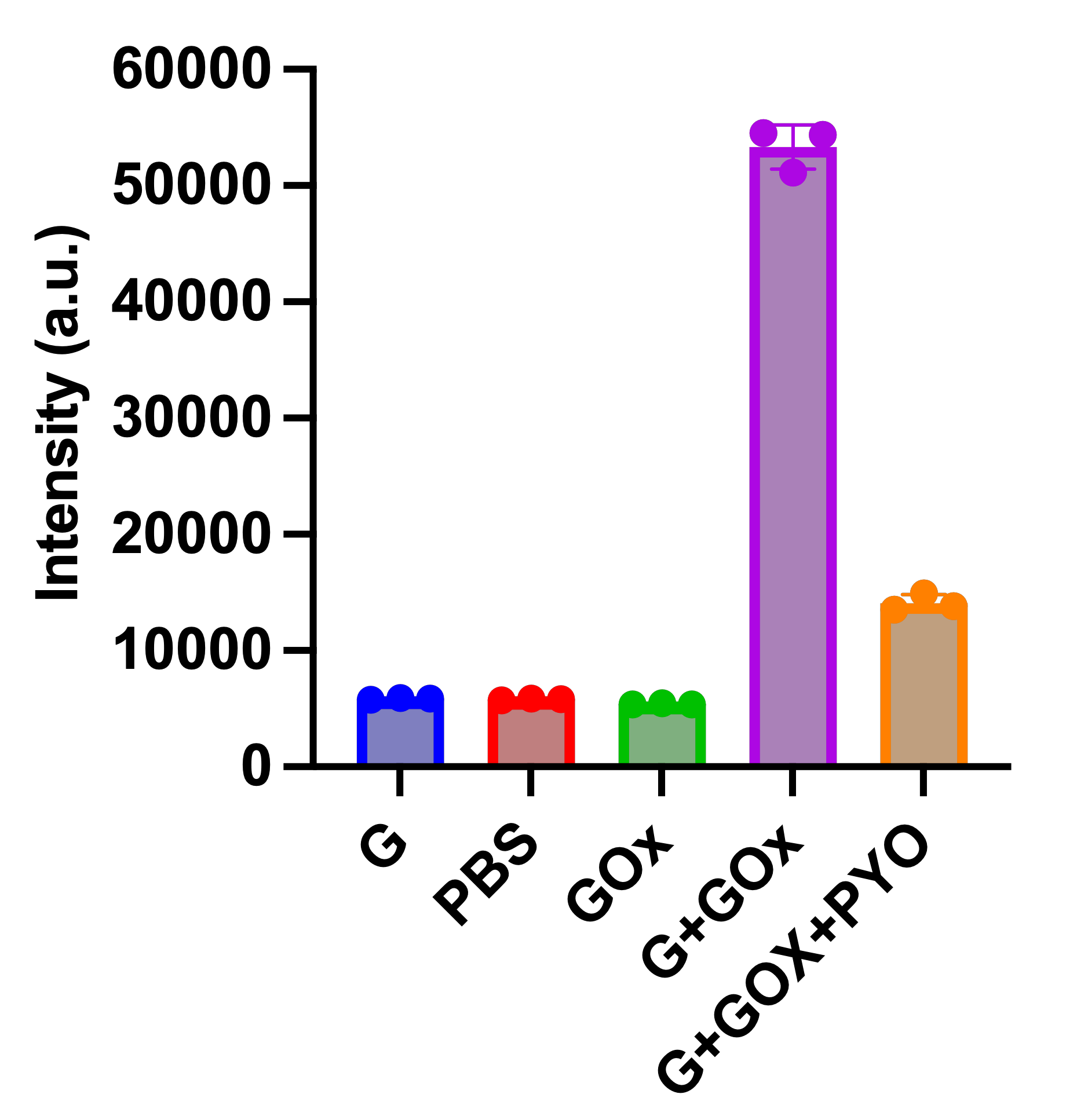

Supplement: AN-151-D6AN00043F-s001 [file AN-151-D6AN00043F-s001.zip › manuscript figure s13.tiff]

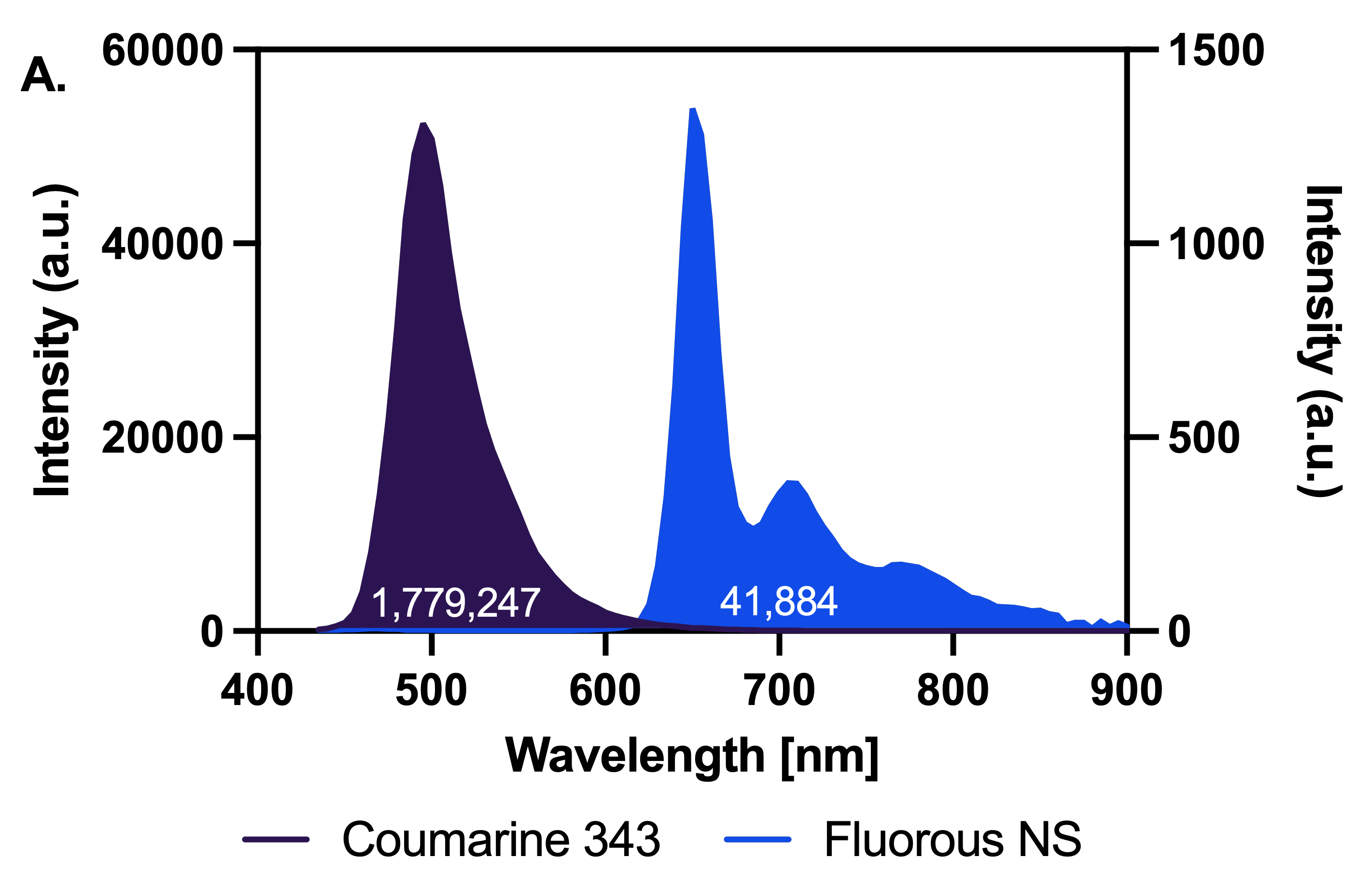

Supplement: AN-151-D6AN00043F-s001 [file AN-151-D6AN00043F-s001.zip › manuscript figure s1a.tiff]

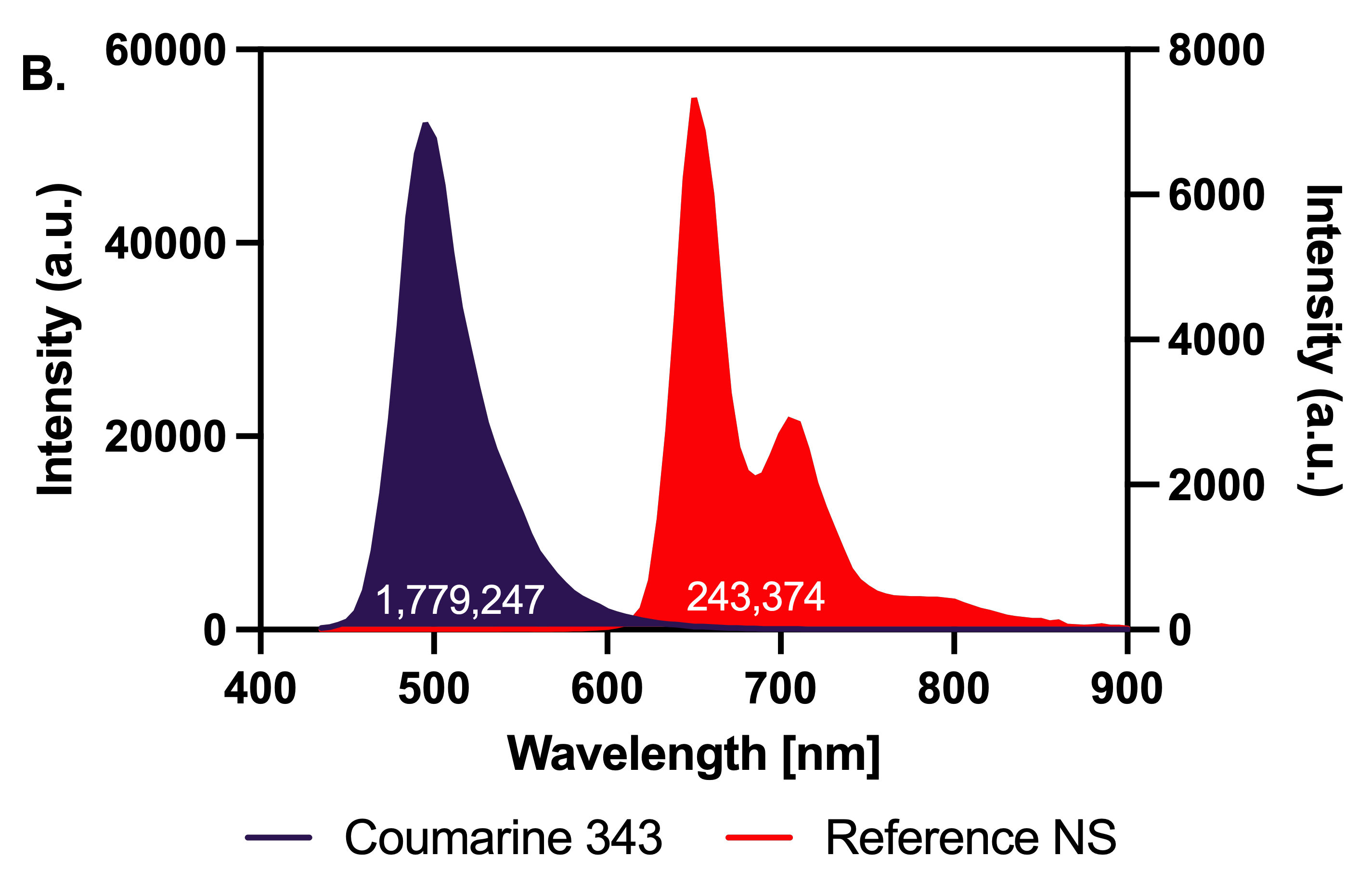

Supplement: AN-151-D6AN00043F-s001 [file AN-151-D6AN00043F-s001.zip › manuscript figure s1b.tiff]

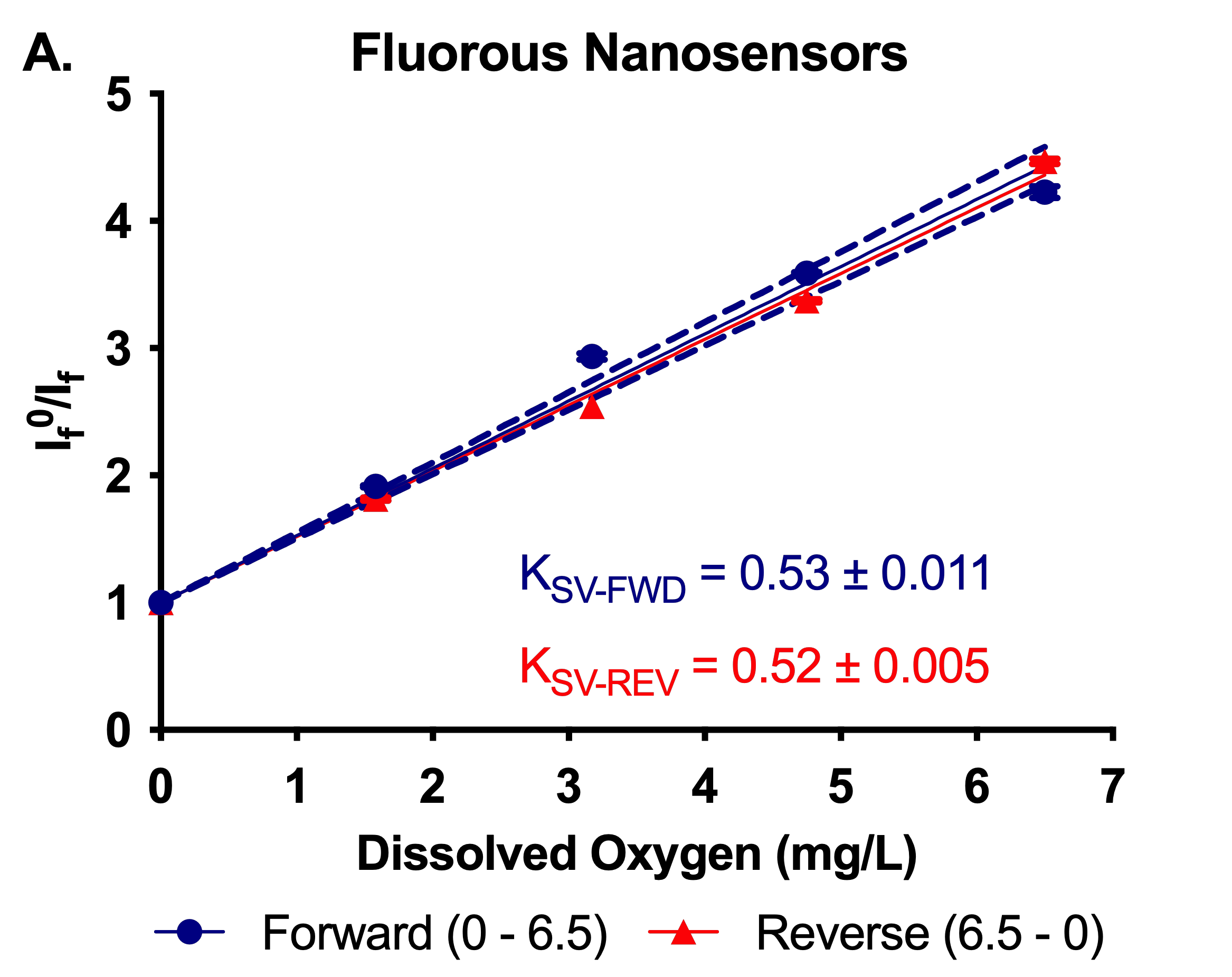

Supplement: AN-151-D6AN00043F-s001 [file AN-151-D6AN00043F-s001.zip › manuscript figure s2a.tiff]

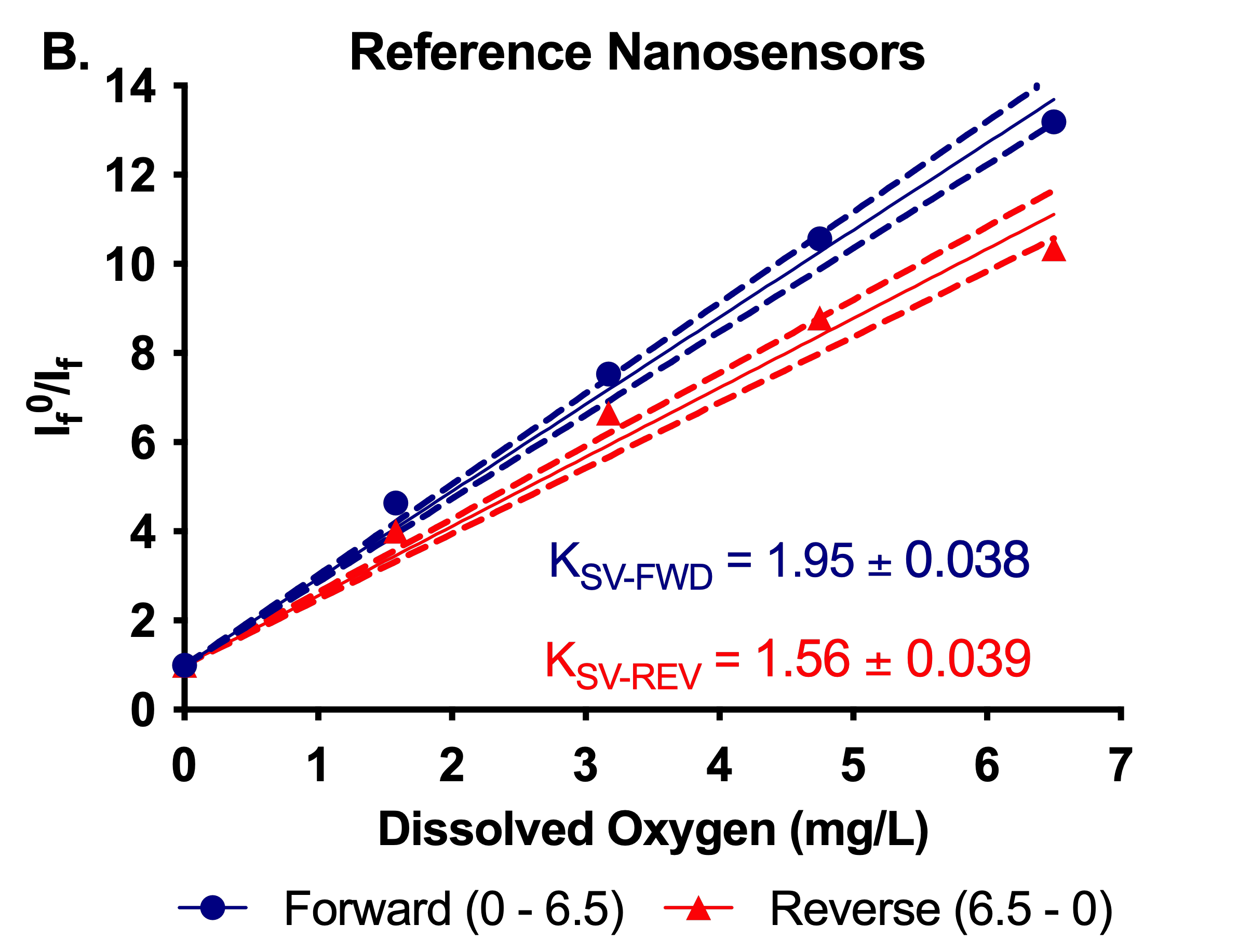

Supplement: AN-151-D6AN00043F-s001 [file AN-151-D6AN00043F-s001.zip › manuscript figure s2b.tiff]

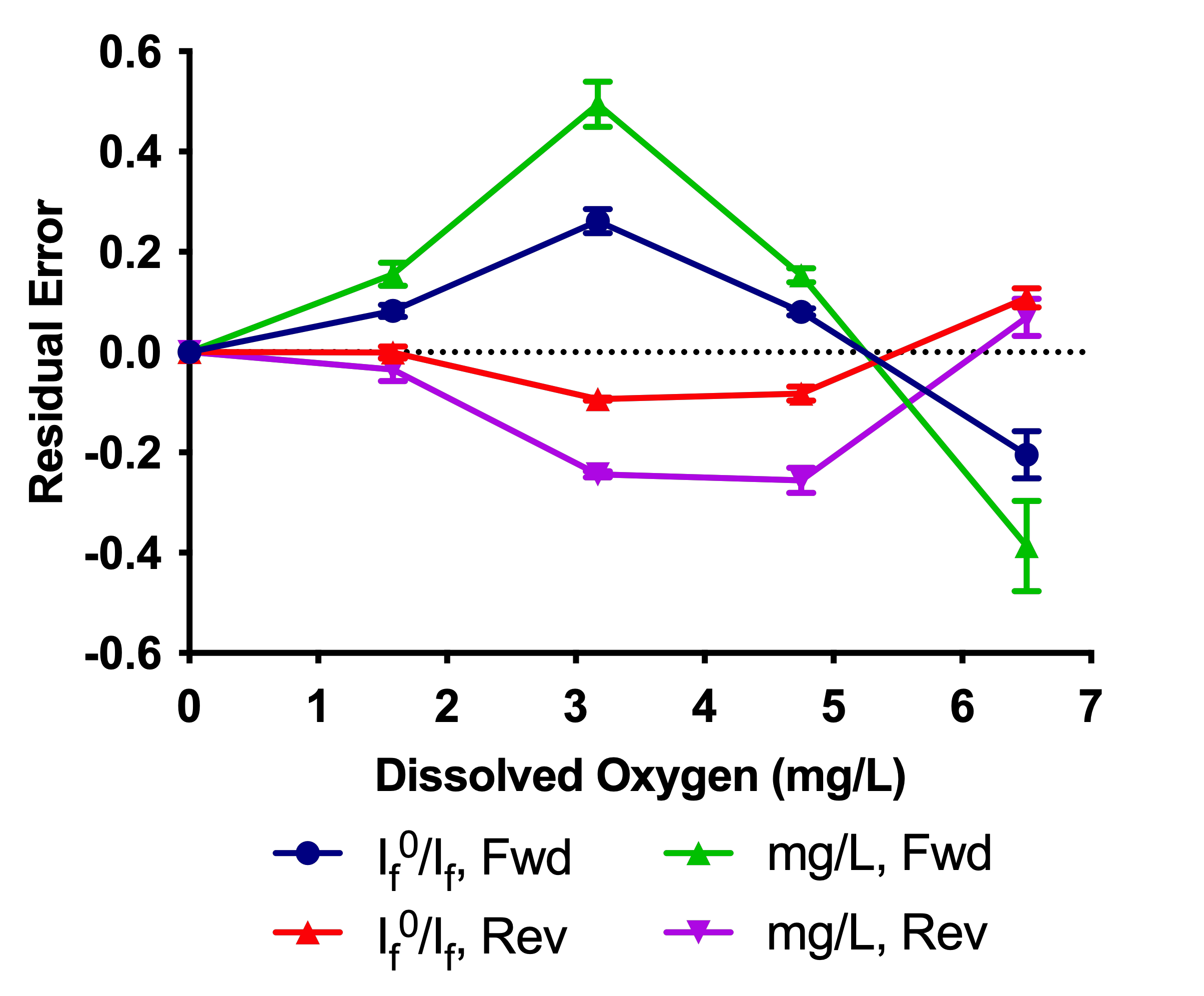

Supplement: AN-151-D6AN00043F-s001 [file AN-151-D6AN00043F-s001.zip › manuscript figure s3.tiff]

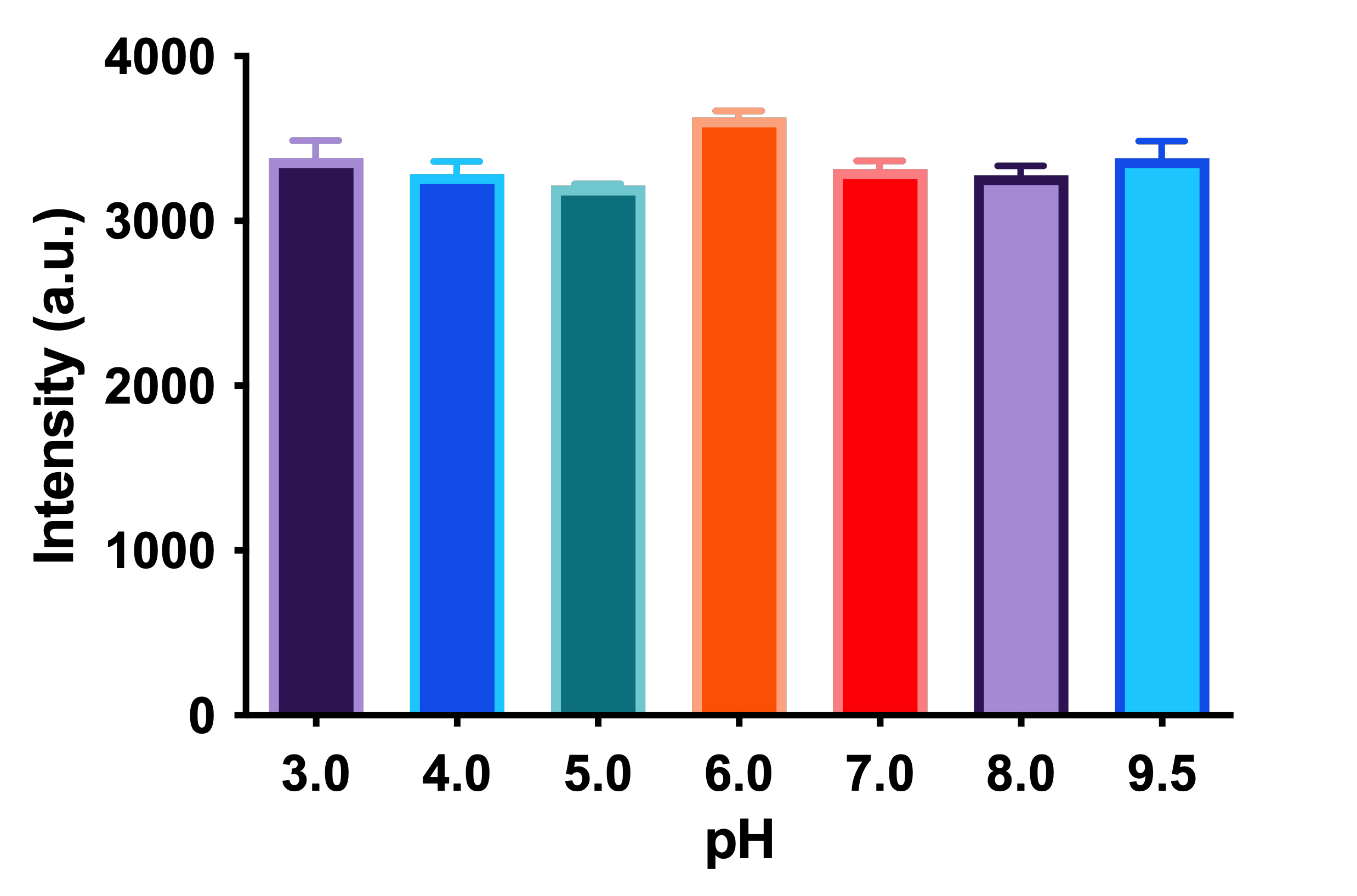

Supplement: AN-151-D6AN00043F-s001 [file AN-151-D6AN00043F-s001.zip › manuscript figure s4.tiff]

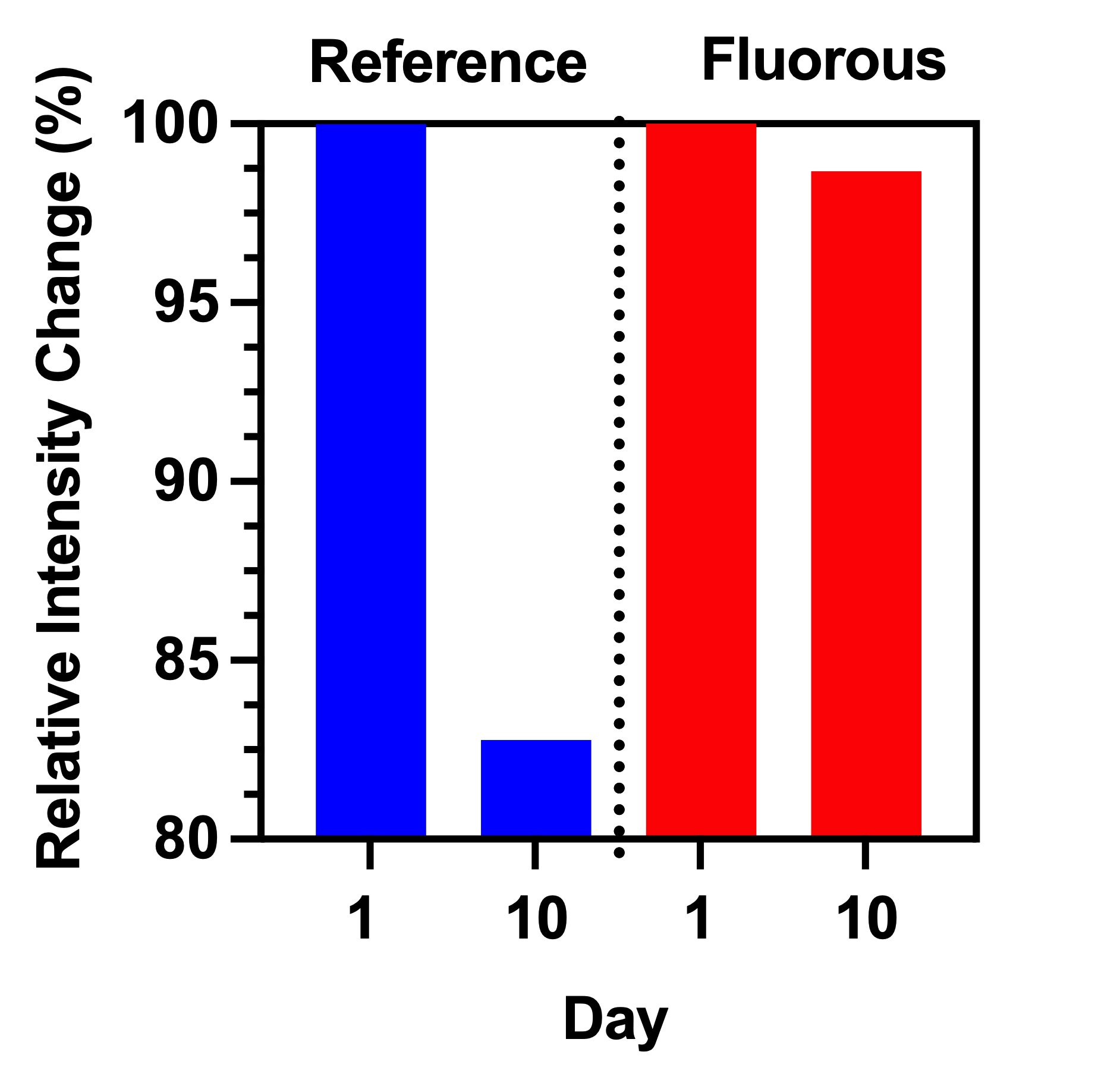

Supplement: AN-151-D6AN00043F-s001 [file AN-151-D6AN00043F-s001.zip › manuscript figure s5.tiff]

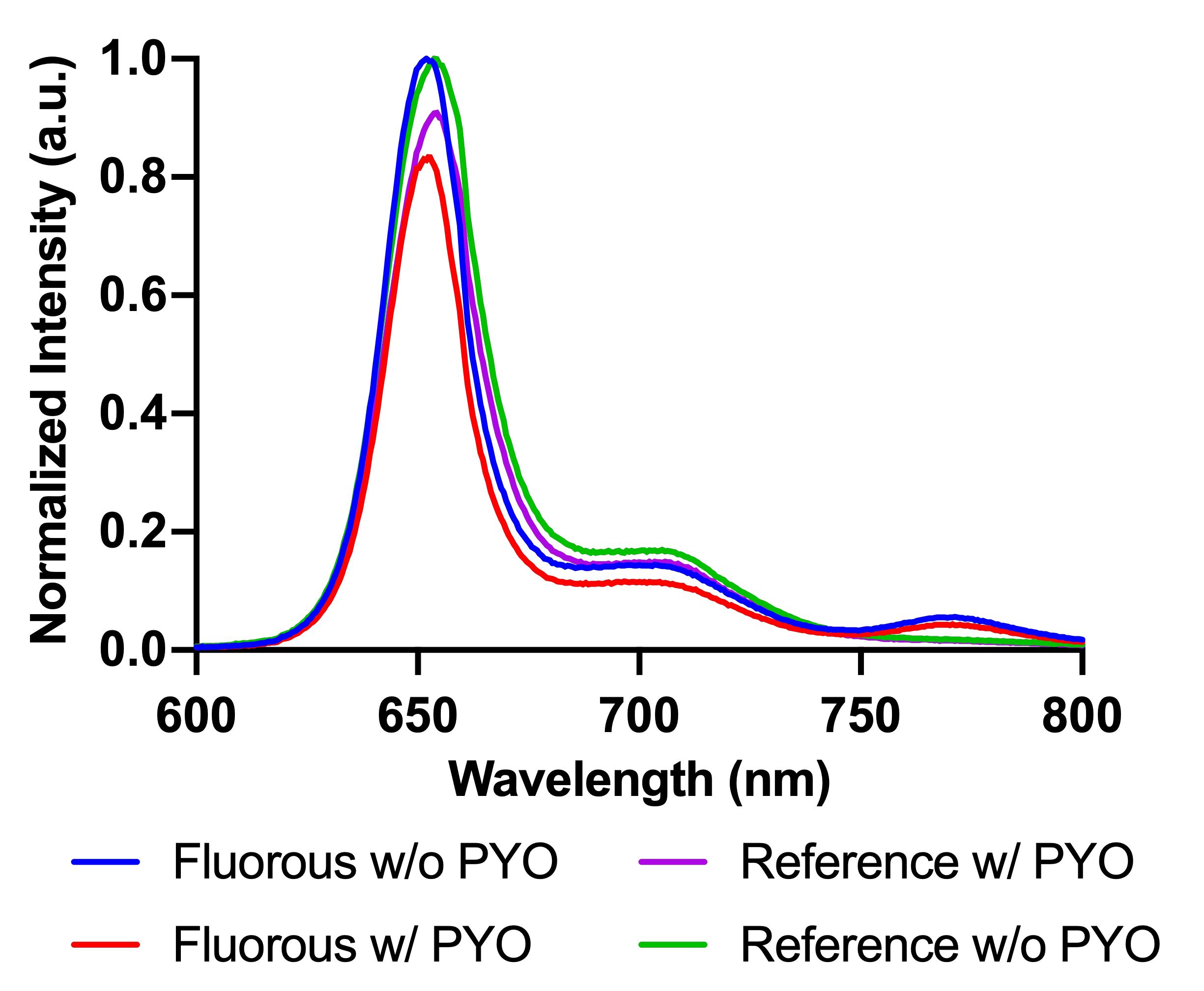

Supplement: AN-151-D6AN00043F-s001 [file AN-151-D6AN00043F-s001.zip › manuscript figure s6.tiff]

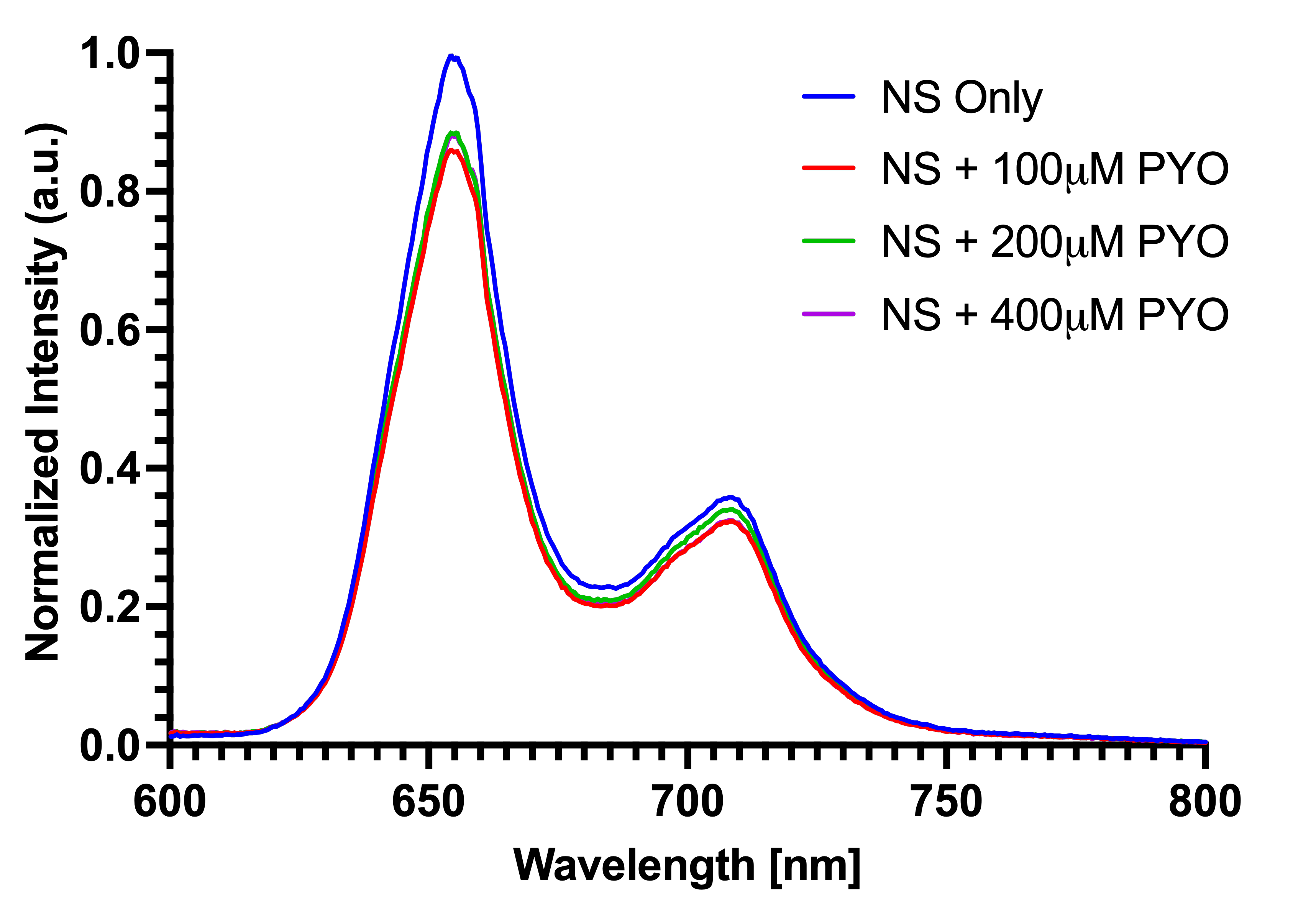

Supplement: AN-151-D6AN00043F-s001 [file AN-151-D6AN00043F-s001.zip › manuscript figure s7.tiff]

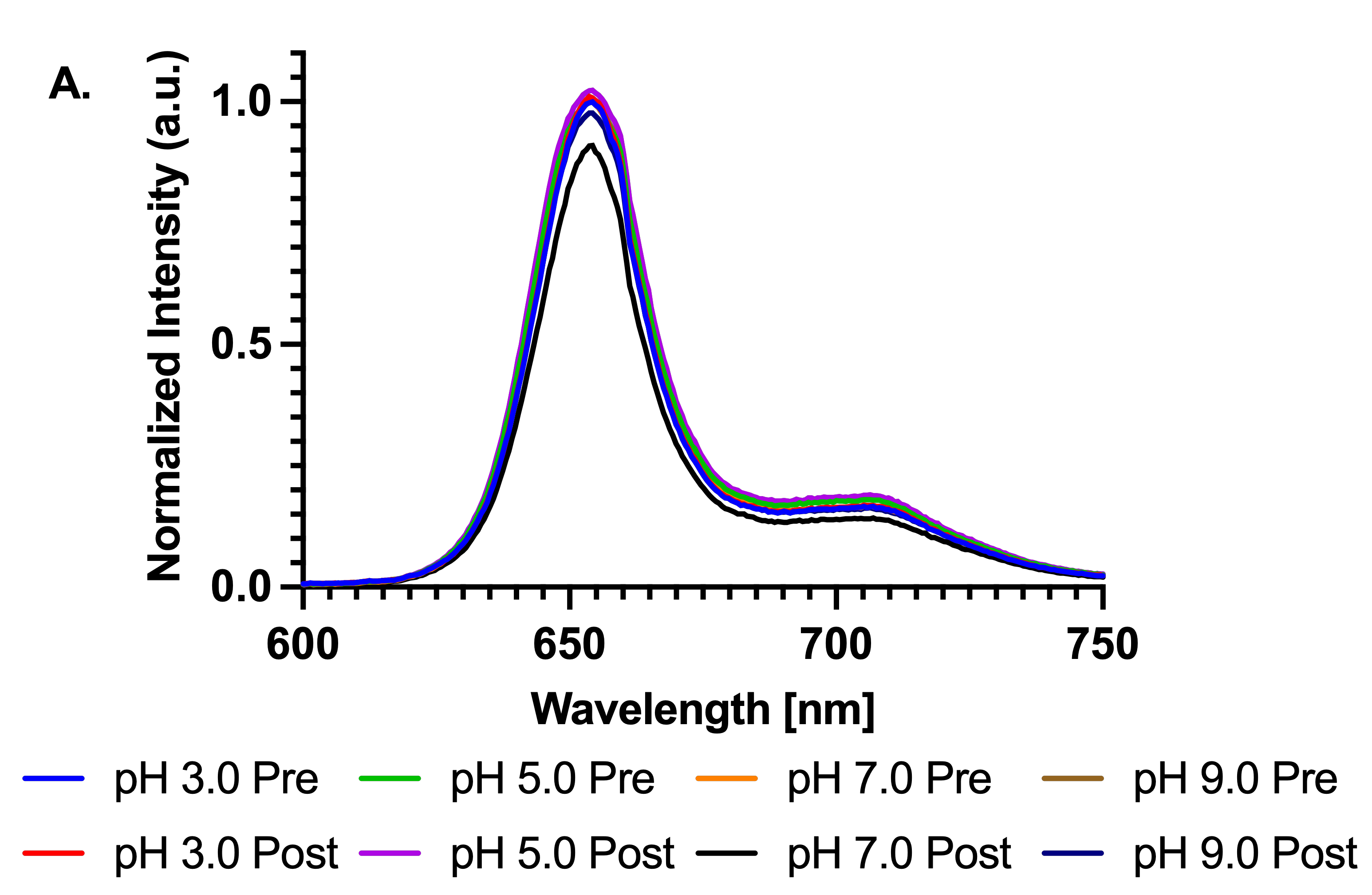

Supplement: AN-151-D6AN00043F-s001 [file AN-151-D6AN00043F-s001.zip › manuscript figure s8a.tiff]

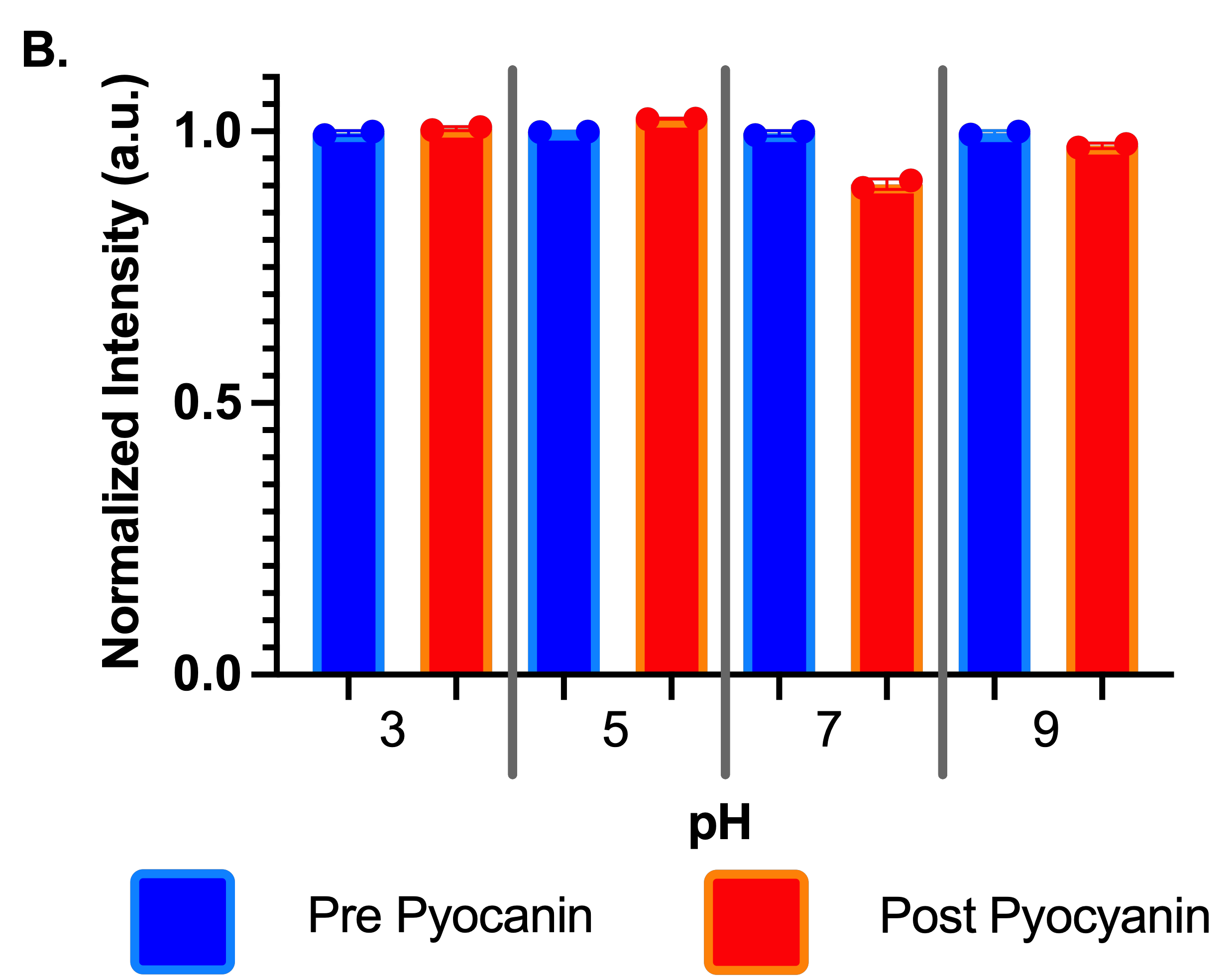

Supplement: AN-151-D6AN00043F-s001 [file AN-151-D6AN00043F-s001.zip › manuscript figure s8b.tiff]

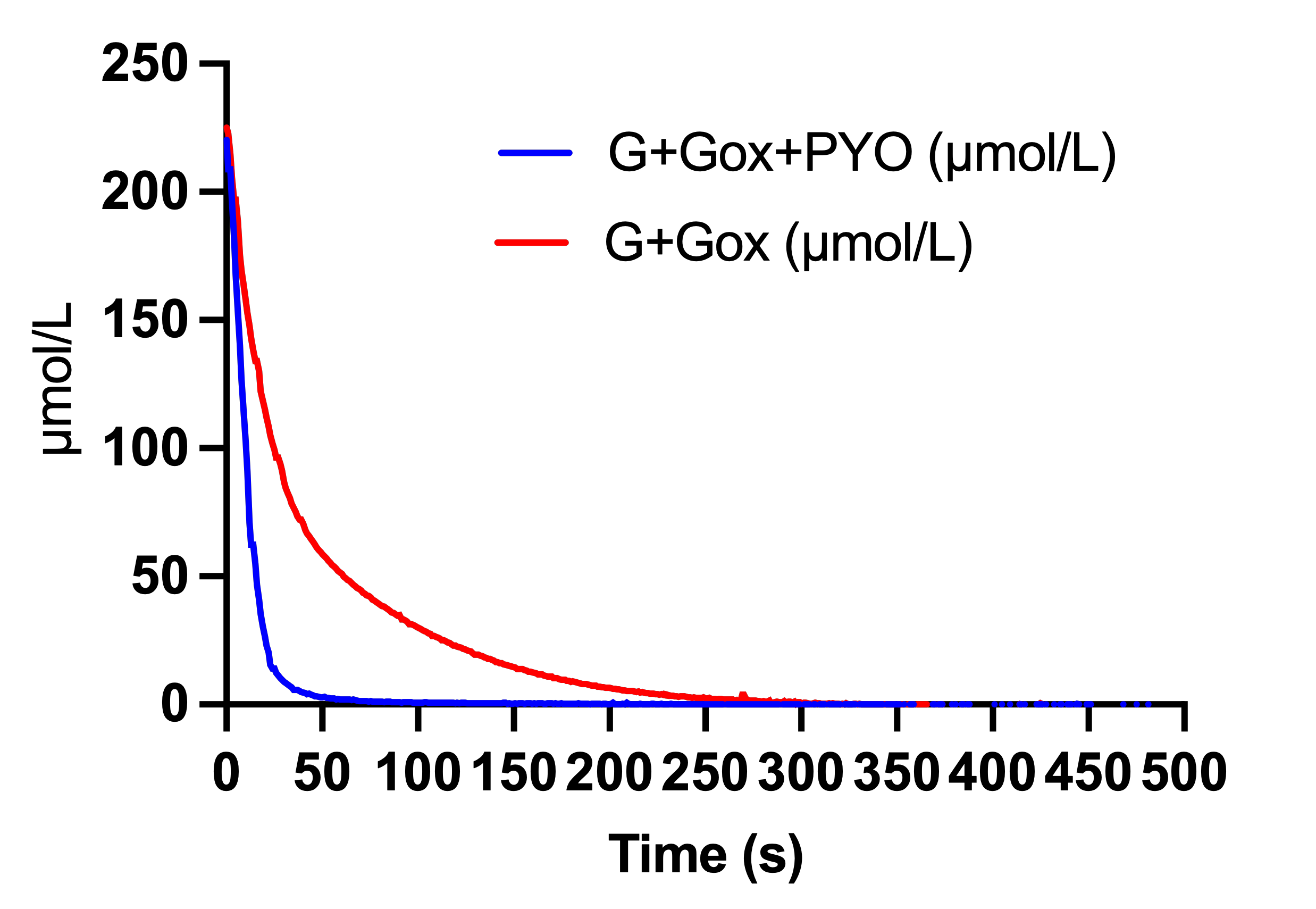

Supplement: AN-151-D6AN00043F-s001 [file AN-151-D6AN00043F-s001.zip › manuscript figure s9.tiff]
